# Early Warning Systems for Acute Respiratory Infections: Scoping Review of Global Evidence

**DOI:** 10.2196/62641

**Published:** 2024-11-07

**Authors:** Atushi Patel, Kevin Maruthananth, Neha Matharu, Andrew D Pinto, Banafshe Hosseini

**Affiliations:** 1 Upstream Lab, MAP Centre for Urban Health Solutions Li Ka Shing Knowledge Institute, St. Michael’s Hospital Unity Health Toronto Toronto, ON Canada; 2 Department of Family and Community Medicine St. Michael’s Hospital Unity Health Toronto Toronto, ON Canada; 3 Department of Family and Community Medicine Faculty of Medicine University of Toronto Toronto, ON Canada; 4 Division of Clinical Public Health Dalla Lana School of Public Health University of Toronto Toronto, ON Canada

**Keywords:** early warning systems, acute respiratory infections, early detection systems

## Abstract

**Background:**

Early warning systems (EWSs) are tools that integrate clinical observations to identify patterns indicating increased risks of clinical deterioration, thus facilitating timely and appropriate interventions. EWSs can mitigate the impact of global infectious diseases by enhancing information exchange, monitoring, and early detection.

**Objective:**

We aimed to evaluate the effectiveness of EWSs in acute respiratory infections (ARIs) through a scoping review of EWSs developed, described, and implemented for detecting novel, exotic, and re-emerging ARIs.

**Methods:**

We searched Ovid MEDLINE ALL, Embase, Cochrane Library (Wiley), and CINAHL (Ebsco). The search was conducted on October 03, 2023. Studies that implemented EWSs for the detection of acute respiratory illnesses were included. Covidence was used for citation management, and a modified Critical Appraisal Skills Programme (CASP) checklist was used for quality assessment.

**Results:**

From 5838 initial articles, 29 met the inclusion criteria for this review. Twelve studies evaluated the use of EWSs within community settings, ranging from rural community reporting networks to urban online participatory surveillance platforms. Five studies focused on EWSs that used data from hospitalization and emergency department visits. These systems leveraged clinical and admission data to effectively detect and manage local outbreaks of respiratory infections. Two studies focused on the effectiveness of existing surveillance systems, assessing their adaptability and responsiveness to emerging threats and how they could be improved based on past performance. Four studies highlighted the integration of machine learning models to improve the predictive accuracy of EWSs. Three studies explored the applications of national EWSs in different health care settings and emphasized their potential in predicting clinical deterioration and facilitating early intervention. Lastly, 3 studies addressed the use of surveillance systems in aged-care facilities, highlighting the unique challenges and needs of monitoring and responding to health threats in environments housing vulnerable populations. The CASP tool revealed that most studies were relevant, reliable, and of high value (score 6: 11/29, 38%; score 5: 9/29, 31%). The common limitations included result generalizability, selection bias, and small sample size for model validation.

**Conclusions:**

This scoping review confirms the critical role of EWSs in enhancing public health responses to respiratory infections. Although the effectiveness of these systems is evident, challenges related to generalizability and varying methodologies suggest a need for continued innovation and standardization in EWS development.

## Introduction

A lack of preparedness and delayed action in response to emerging infectious diseases, particularly acute respiratory infections (ARIs), have caused a significant strain on hospital services globally, despite countries adopting various risk mitigation measures [1]. ARIs, such as influenza, pneumonia, and the more recent COVID-19, impose a significant burden on health care systems due to their potential to spread rapidly and cause severe illness, especially among vulnerable populations [2]. In such circumstances, tools that can facilitate the early identification of symptom surges are crucial for allocating hospital resources efficiently and ensuring that patients receive the best care, ultimately preventing health systems from being overwhelmed [1,3].

Early warning systems (EWSs) are pivotal in this regard. An EWS is a structured approach designed to detect early signs of clinical deterioration in patients, potential outbreaks, or public health threats, enabling timely intervention [4,5]. These systems rely on the continuous collection, integration, and analysis of various data sources, including clinical observations, laboratory results, epidemiological data, and other relevant health information [4,5]. EWSs typically incorporate algorithms or scoring systems that trigger alerts when specific criteria are met, signaling the need for immediate medical or public health response [6,7]. In a clinical setting, EWSs can monitor vital signs and other patient indicators to identify patterns consistent with an increased risk of deterioration. For example, the National Early Warning Score (NEWS) system used in the United Kingdom assigns a score to patients based on their vital signs, with higher scores indicating a higher risk of adverse outcomes, such as sepsis or cardiac arrest [8]. This allows health care providers to intervene early. On a broader scale, EWSs can be integrated into public health surveillance systems to monitor and detect infectious disease outbreaks. These systems, such as the one operated by the Korea Disease Control and Prevention Agency (KDCA), use a combination of real-time data from hospitals, laboratories, and other health institutions to identify unusual patterns that may indicate the emergence of a new infectious disease or the resurgence of an existing one [9]. Similarly, the European Centre for Disease Prevention and Control (ECDC) coordinates the Early Warning and Response System (EWRS), which facilitates the exchange of information among EU Member States to quickly respond to public health threats [10]. In Canada, various surveillance systems, such as FluWatch and Alberta’s TARRANT program, aim to monitor the spread of influenza and provide up-to-date information on flu activity to ensure timely interventions, particularly during flu seasons [11,12]. However, the effectiveness of these systems has faced criticism during the COVID-19 pandemic for various reasons, including a decline in participating sentinel primary care practices, which can affect active surveillance [13,14].

Given the global nature of ARI threats and the challenges faced in their detection and management, it is crucial to evaluate the effectiveness of surveillance systems and EWSs specifically tailored to ARIs in various countries. While there are existing reviews on EWSs for various conditions, there is a notable gap in the literature specifically focusing on ARIs. Therefore, this scoping review aims to provide a detailed synthesis and evaluation of the existing evidence on surveillance systems, with a focus on their application in detecting and managing ARIs. The review will explore the advantages, disadvantages, limitations, and potential applications of EWSs in the context of ARIs.

## Methods

### Overview of Study Methods

This scoping review was registered with Open Science [15] and was guided by the PRISMA-ScR (Preferred Reporting Items for Systematic Reviews and Meta-Analyses extension for Scoping Reviews) checklist (Multimedia Appendix 1) [16]. Further guidance was sought by reviewers to guide the formation of the research question, identification of relevant criteria for the topic of choice, screening and extraction of the literature, and writing of this scoping review [17,18].

### Search Strategy and Eligibility Criteria

**Figure 1:**
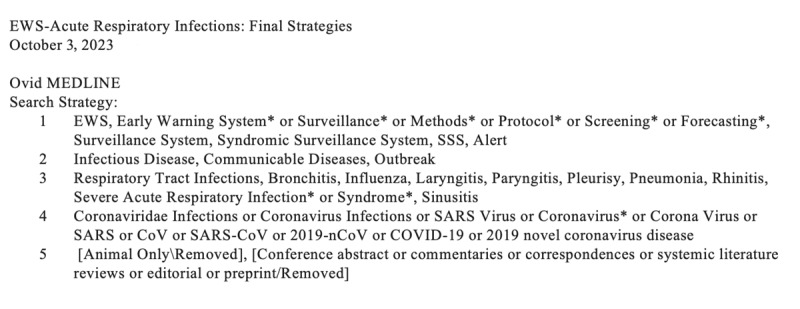
Example of the search strategy for the Ovid MEDLINE database.

An information specialist (Katinka English) developed and refined the search strategies through an iterative process in consultation with the review team. The MEDLINE strategy was reviewed by the study team prior to execution using the Peer Review of Electronic Search Strategies (PRESS) checklist. We searched Ovid MEDLINE ALL, Embase Classic + Embase, Cochrane Library (Wiley), and CINAHL (Ebsco) on October 03, 2023. The search included literature from inception to October 2023, with no limitations on the language of the available evidence or the population examined. No additional grey literature search was conducted. The search strategies combined controlled vocabulary and keywords related to EWSs, syndromic surveillance systems, and communicable diseases, with no language restrictions. We excluded animal-only records, opinion pieces, case studies, conference abstracts, and other irrelevant publication types as we were seeking studies reporting original data only. Clinical studies, including randomized controlled trials, cohort studies, and cross-sectional studies, were included, and all historical data were considered. EWSs and any comparators were explored, with outcomes including the detection of novel, exotic, or re-emerging ARIs. Figure 1 summarizes the terms used to conduct the search. Records were downloaded and deduplicated using EndNote version 9.3.3 (Clarivate Analytics) and uploaded to Covidence (Veritas Health Innovation) for efficient data management, screening, and synthesis. In addition, the reference lists of retrieved articles and relevant systematic reviews were searched to identify other relevant studies.

The eligibility criteria for study inclusion were as follows: (1) clinical (human-based) studies using EWSs for detecting ARIs and (2) studies reporting original data. We did not limit the inclusion criteria based on study design to capture all available evidence. The exclusion criteria were as follows: (1) animal studies; (2) studies not focused on EWSs for ARIs; and (3) reviews, systematic reviews, opinions, editorials, and case reports.

### Data Collection and Quality Assessment

The search was conducted on October 03, 2023, and included eligible evidence from inception to the date of the search. Covidence, a web-based literature review software [19], was used to manage citations throughout the review. A team of reviewers (AP, Hibah Sehar, Krishihan Sivapragasam, and Tina Vosoughi) was trained to screen citations according to the eligibility criteria. To ensure interrater reliability, each reviewer was trained on a set of 10 citations and then independently screened an additional 10 citations. Discrepancies were discussed and resolved following the independent screening of the training citation set. Title and abstract screening for each citation was performed independently by 2 reviewers. Any discrepancies during the screening process were resolved through discussion or by a third reviewer (AP). Full-text screening was conducted by 2 reviewers (AP and Hibah Sehar) for studies that passed the initial screening.

Data extraction was conducted using Covidence, with 3 team members (AP, KM, and NM) performing the extraction independently. Data items to be extracted were determined by the eligibility criteria and included information on the population studied, the details of the EWS or intervention used, any comparators, and the outcomes examined, along with strengths and limitations. To ensure thoroughness, a single study was extracted by 1 reviewer (AP) and shared with the study team to confirm that all relevant information was captured. The following data were extracted: authors, title, journal, year, funding source, study design, study population, country, demographics, sample size, intervention, description of the EWS, methodology for EWS implementation, reasons for intervention (development, description, and implementation), outcomes of interest (type of EWS, features used, effectiveness, and accuracy), comparator (yes or no; if yes, description), the total number for the intervention, the total number for the comparator, duration of the intervention, results, limitations, strengths, implications for future research, and conclusions. Missing information from any article was noted. To ensure interrater reliability, each reviewer was trained to extract 1 article and then independently extracted data from 2 additional articles. Discrepancies were discussed and resolved following these independent extractions. One team member (AP) checked each extraction for validity and completeness.

The methodological quality of eligible studies was independently assessed by 3 reviewers (AP, NM, and Krishihan Sivapragasam) using an adapted 6-question Critical Appraisal Skills Programme (CASP) checklist [7], focusing on research objectives, methodology, data reproducibility, comparability, and outcome ascertainment. Data were categorized in various forms, including but not limited to the types of EWSs used in studies, institutions that implemented them, and target populations for their use.

### Data Synthesis

We employed a narrative synthesis approach to review and summarize the objectives, EWSs used, and clinical relevance of each study. Studies were organized based on the methods used in the development or implementation of the EWS. Information on the EWS, including features used, effectiveness, and accuracy, was extracted and synthesized.

## Results

### Study Selection and Characteristics

**Figure 2:**
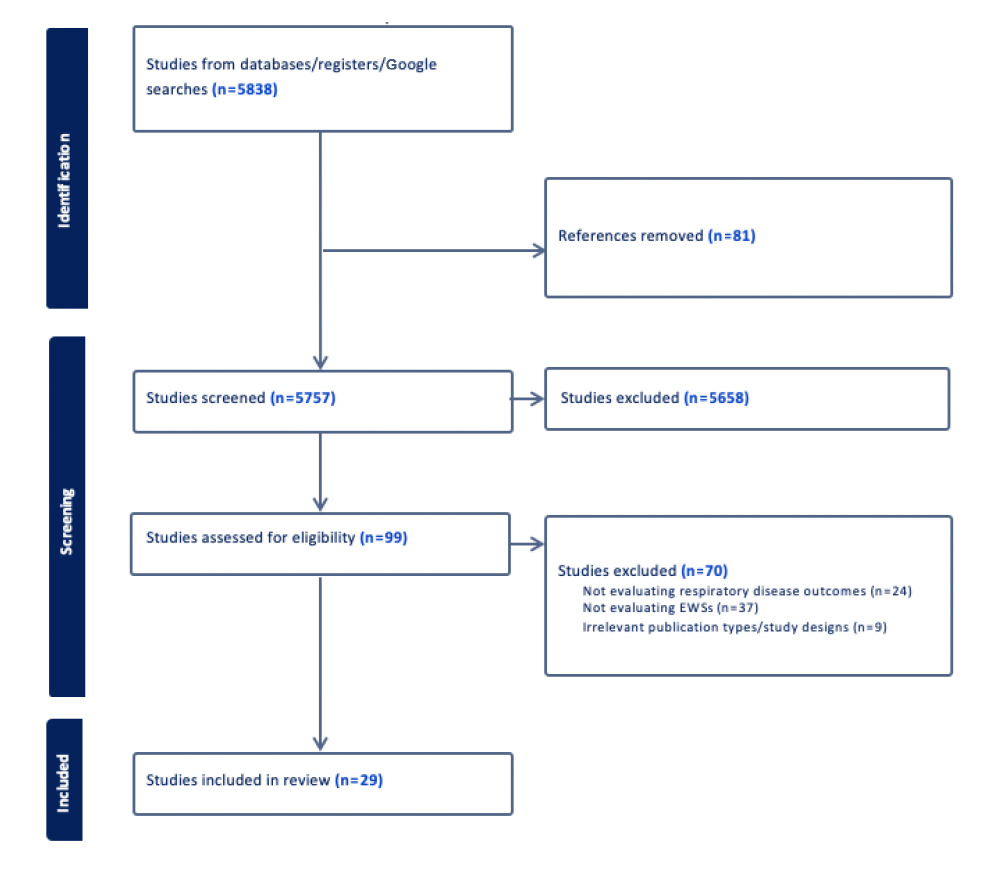
Selection process of eligible studies from all identified citations. EWS: early warning system.

The initial screening yielded 5838 articles, from which 81 duplicate records were removed using EndNote. After primary title and abstract screening, 5658 studies were excluded as they did not meet the eligibility criteria, leaving 99 for further evaluation. Secondary full-text screening resulted in the exclusion of 70 studies, leaving a total of 29 articles that met our inclusion criteria for this review on EWSs used in the detection of ARIs. The selection process is presented in Figure 2.

Geographical settings varied across the studies, with 5 studies conducted in the United States [20-24], 3 in Australia [25-27], 2 in Italy [28,29], 2 in China [30,31], 2 in the Netherlands [32,33], 2 in Germany [34,35], 2 in Ghana [36,37], and 1 each in Canada [38], India [39], Senegal [40], Singapore [41], Brazil [42], Spain and Argentina [43], Scotland [44], Spain [45], the Pacific Islands [46], Japan [47], and South Korea [48].

In our review, the studies were categorized into 6 primary areas based on the focus of their surveillance systems. These categories included community-based surveillance systems, hospital- and emergency department (ED)-based systems, EWSs for institutionalized elderly people, EWSs based on the machine model, previously implemented systems, and the NEWS. The distribution of studies across these categories is summarized in Table 1.

**Table 1:** Categorization of studies by surveillance system focus.

Category	Studies
Community-based surveillance systems	Seck et al [40], 2023; Lee et al [38], 2021; Ferreira et al [42], 2022; Awekeya et al [37], 2021; Katayama et al [47], 2020; Kavanagh et al [44], 2012; Dong et al [31], 2016; Nuvey et al [36], 2019; Mostashari et al [23], 2003; Schrell et al [45], 2013; van den Wijngaard et al [33], 2008; Kool et al [46], 2012
Surveillance systems using hospital and emergency department data	Lukowsky et al [24], 2022; Hong et al [48], 2022; Cashmore et al [27], 2013; Buda et al [35], 2017; van den Wijngaard et al [32], 2010
Application of surveillance systems for institutionalized elderly people	Rosewell et al [25], 2010; Gugliotta et al [28], 2021; Quinn et al [26], 2023
Application of machine learning and algorithms in the development of early warning systems	Jakob et al [34], 2022; Chang et al [30], 2022; El Halabi et al [21], 2022; Alavi et al [20], 2022
Application of previously implemented surveillance systems	Yohannes et al [22], 2023; Htun et al [41], 2020
Application of the National Early Warning Score (NEWS)	Khuraijam et al [39], 2022; Klén et al [43], 2023; Tagliabue et al [29], 2021

### Implementation of EWSs Across Community, Hospital, and Institutional Settings

#### Applications of Community-Based Surveillance Systems

A total of 12 studies (41%) evaluated the use of community-based EWSs for ARIs (Table 2). These studies assessed surveillance systems utilizing telephone calls, online platforms, ambulance dispatch data, and other clinical data across various countries and regions. The primary objective was often early detection to prevent the worsening of health care outcomes and to reduce the burden on health care systems within communities.

Seck et al [40] examined the implementation of a community event–based surveillance (CEBS) system in Senegal to facilitate timely detection and response to potential COVID outbreaks [40]. The system used telephone calls from community members to report symptomatic individuals or contacts with confirmed COVID cases. Medical personnel followed up on suspected cases within 24 hours [40]. Of the 10,751 COVID-specific calls received, 50.2% were referred to health districts for further investigation, with 25% confirmed as positive COVID cases [40].

Lee et al [38] evaluated the use of FluWatchers in Canada, an online participatory syndromic surveillance platform designed to enhance influenza-like illness (ILI) surveillance. FluWatchers demonstrated higher reliability, accuracy, and usefulness compared to the Sentinel Practitioner ILI Reporting System (SPIR), with season-over-season retention of 80% of participants [38]. Furthermore, the data showed a strong correlation with laboratory-confirmed influenza cases across 4 seasons [38]. Ferreira et al [42] reported on the implementation of a public health surveillance system for COVID in Serrana, Brazil, focusing on detection, tracing, and patient support through a network of community institutions. The enhanced surveillance system resulted in a decrease in the positivity rate of SARS-CoV-2 from 36.7% before implementation to 26.1% after (*P*<.001) [42].

Awekeya et al [37] evaluated the effectiveness of Ghana’s COVID surveillance system in the New Juaben South Municipality. The system was found to be moderately sensitive (55.6%), with a predictive value positive (PVP) of 31.3% [37]. Similarly, another study [36] evaluated the effectiveness of the surveillance system for ILI in the Greater Accra region in Ghana, reporting a PVP ranging from 4.7% to 14.8%, with a median of 7.4%.

Katayama et al [47] determined the association between the number of influenza patients and telephone triages in Osaka, Japan. Of the 1,065,628 telephone triages recorded, 101,572 were for a fever and 465,971 were identified as influenza cases [47]. Their analysis using a linear regression model showed a high correlation, with an R^2^ of 0.832 and a Spearman correlation coefficient of 0.923 (*P*<.001), indicating a strong positive relationship between telephone triages for fever and the number of influenza cases [47]. Likewise, Kavanaugh et al [44] observed a telephone service in Scotland and examined its ability to predict the start of the influenza season. Comparison of the service for colds and flu calls in relation to clinician-reported rates showed that the system was able to predict influenza outbreaks approximately 1 week prior to clinician-reported rates, thus providing some degree of early warning [44].

**Table 2:** Summary of studies applying community-based surveillance systems.

Study	Study design	Country	Sample size (n)	Study population	Outcomes	Objectives	Findings	Limitations
Seck et al [40], 2023	Cross-sectional (descriptive analysis)	Senegal	10,760	—^a^	Effectiveness	Implement the CEBS^b^ system for timely detection and response to potential COVID-19 cases, contributing to the overall surveillance efforts in the country.	Of the COVID-19 calls received by the alert system, 50.2% were validated and sent for further investigation, of which only 1354/5402 (25%) were positive COVID cases.	Self-reported data; hence, over/under reporting; Lacks generalizability
Lee et al [38], 2021	Cross-sectional (serial)	Canada	6827 (n=505 from 2015-2016; n=998 from 2016-2017; n=2114 from 2017-2018; n=3210 from 2018-2019)	—	Effectiveness and accuracy	Evaluate the FluWatchers program on acceptability, reliability, accuracy, and usefulness for the detection of influenza-like illnesses.	FluWatchers presented season-over-season retention of 80% of participation. Percentage of weeks where the number of FluWatchers participants was within ±5%, ±10%, or ±15% from the season median was higher than SPIR^c^. FluWatchers data on influenza compared to laboratory-confirmed influenza showed a strong correlation (*P* values of .0036 for 2015-2016, .0029 for 2016-2017, <.0001 for 2017-2018, and .0001 for 2018-2019).	Selection bias; Generalizability
Ferreira et al [42], 2022	Prospective	Brazil	6155 participants (6728 samples)	91.1% were adults and 8.5% were children; median age was 35.0 years	Effectiveness and accuracy	Implement the enhanced surveillance system based on detection, tracing, and patient support for COVID-19.	Of the 6728 samples collected from 6155 participants versus 2770 samples collected in a similar period before, SARS-CoV-2 RNA was detected in 1758 (26.1%) swabs versus 1117 (36.7%) before the implementation of the surveillance system (*P*<.001). Positivity rates varied widely between epidemiological weeks 35/2020 and 5/2021 (IQR 12.8%-31.3%).	Generalizability
Awekeya et al [37], 2021	Cross-sectional (mixed methods descriptive)	Ghana	1090 suspected COVID-19 cases detected; 1959 expected COVID-19 cases detected (NJS municipality hospitals)	—	Effectiveness and accuracy	Evaluate Ghana’s COVID-19 surveillance system to identify if the system aids in the detection of COVID-19.	The system was deemed as moderately sensitive at 55.6%, and the PVP^d^ of the model, defined as the proportion of reported cases that had the diseases detected, was 31.3%.	Generalizability; Did not capture the perspectives of community members on the system
Katayama et al [47], 2020	Retrospective	Japan	1,065,628 telephone triages	0-100 years old; Male: 511,267 or 48.0%; Female: 553,000 or 51.9%; Unknown: 961 or 0.1%	Effectiveness and accuracy	Examine the relationship between the number of telephone triages for fever and the number of influenza patients in Osaka, Japan to predict if this predictive model can control the spread of influenza.	Of the 1,065,628 telephone triages, 101,572 were for a fever, with 465,971 patients with influenza. The adjusted weekly incidence of influenza patients and number of telephone triages for fever showed an R^2^ of 0.842 and a Spearman rank-order coefficient of 0.932. The predicted number of influenza patients in the influenza outbreak season from the linear regression model and the weekly number of influenza patients from December to April showed an R^2^ of 0.832 and a Spearman correlation coefficient of 0.923 (*P*<.001).	Not an official clinical diagnosis of influenza; Selection bias; Presence of confounding factors
Kavanagh et al [44], 2012	Prospective	Scotland	849 calls	All ages	Effectiveness	Assess how the system can be used to identify the start of the influenza season.	Comparison of signals from the service on colds and flu calls in relation to clinician consultation rates showed that the system signaled about 1 week prior to a major rise in clinician rates.	Only able to be implemented in small institutions; Doubt for large institutions
Dong et al [31], 2016	Retrospective	China	783 laboratory-confirmed influenza cases	All ages	Effectiveness and accuracy	Determine which sources of syndromic surveillance (ie, OTC^e^ drug sales, hospital- and school-based ILI^f^, and Baidu search queries) had the strongest correlation with laboratory-confirmed influenza activity.	Syndromic data for hospital ILI%, OTC sales, and school-based ILI correlated well with laboratory data (r=0.732, 0.490, and 0.693, respectively; *P*<.05). Baidu searches for “influenza,” “cough,” and “fever” correlated best with laboratory-confirmed activity; queries for “fever” were the strongest (r=0.924; *P*<.001).	Bias regarding OTC sales; Limits in access to school-based data due to holidays and weekends
Nuvey et al [36], 2019	Retrospective	Ghana	2948	All age groups (median age: 30); Female: 1775 or 60%	Features used, effectiveness, and accuracy	Evaluate the ILI surveillance system in the Greater Accra region, Ghana, to assess the system’s attributes and its performance on set objectives.	PVP of 7.4% (range 4.7%-14.8%); Simplicity score: 1/3; Flexibility score: 3/3; Data quality score: 2/3; Acceptability score: 2/3; Representativeness score: 3/3; Timeliness score: 3/3; Stability score: 2/3.	Prospective application of the system; Relatively low sample size
Mostashari et al [23], 2003	Retrospective (years 1993 to 1998); Prospective (years 1999 to 2003)	United States	—	All ages	Effectiveness and accuracy	Compare the timing of increases in ambulance dispatches to influenza activity.	71 (3.2%) alarms at the 99% level. Of these alarms, 64 (90%) occurred shortly before or during a period of peak influenza in each of the 6 influenza seasons.	Systems are dependent on the availability of population-wide electronic data.
Schrell et al [45], 2013	Retrospective	Spain	2010-2011 season: n=360; 2011-2012 season: n=283	All ages	Effectiveness and accuracy	Assess the local implementation of syndromic surveillance.	With the best algorithm settings, we achieved 70%/63% sensitivity and 89%/95% specificity for 2010-11/2011-12.	Analysis has been conducted retrospectively; Objectivity of physicians in diagnosing ILI
van den Wijngaard et al [33], 2008	Retrospective	Netherlands	—	All ages	Effectiveness	Use 6 different types of health care data for syndromic surveillance of respiratory disease.	Syndrome time series in all registries correlated strongly with *S. pneumoniae* (hospital: r=0.73, GP^g^: r=0.71, mortality: r=0.56, pharmacy: r=0.75, laboratory submissions: r=0.58, absenteeism: r=0.69). Hospital (r=0.74 and r=0.57), GP (r=0.67 and r=0.61), pharmacy (r=0.58 and r=0.60), and laboratory submission (r=0.53 and r=0.47) data were strongly correlated with RSV^h^ and influenza A counts. Mortality data correlated strongly with influenza A (r=0.65) and influenza B (r=0.50) infections.	Short duration of the time series, especially for absenteeism and pharmacy data; therefore, whether our observed associations between syndromes and pathogen counts can be generalized remains unclear.
Kool et al [46], 2012	Prospective	Pacific Island countries and territories	—	All ages	Effectiveness	Implementation of simplified surveillance was proposed, with case definitions based on clinical signs and symptoms without the need for laboratory confirmation or information on symptoms, location, sex, and age.	Two peaks in ILI cases in Fiji were later confirmed in the laboratory as influenza. Acute fever and rash: 13; Diarrhea: 173; ILI: 596; Prolonged fever: 24.	Short duration (1 year)

^a^Not applicable.

^b^CEBS: community event–based surveillance.

^c^SPIR: Sentinel Practitioner Influenza-Like Illness Reporting System.

^d^PVP: predictive value positive.

^e^OTC: over-the-counter.

^f^ILI: influenza-like illness.

^g^GP: general practitioner.

^h^RSV: respiratory syncytial virus.

In a unique study, Dong et al [31] investigated 4 sources of syndromic surveillance: over-the-counter drug sales, search queries from a Chinese internet service called Baidu, and ILI data from hospitals and schools. The study aimed to identify the most effective source for detecting influenza activity in Tianjin, China, by comparing each source’s correlation with laboratory-confirmed influenza activity [31]. Syndromic data from over-the-counter sales, and hospital and school-based ILI showed significant correlations with laboratory data (r=0.490, 0.732, and 0.693, respectively; *P*<.05), while Baidu searches for “fever” showed the highest correlation (r=0.924; *P*<.001) [31]. Notably, school-based absence reporting detected influenza virus activity 1 week earlier than laboratory confirmation [31]. Likewise, van den Wijngaard et al [33] used 6 data types, including hospitalization, mortality, diagnostic test requests, pharmacy dispensations, absenteeism, and general practice consultations, to detect emerging respiratory outbreaks [33]. Laboratory diagnostic test request data were strongly correlated with respiratory syncytial virus (RSV) (r=0.53) and influenza A counts (r=0.47), while mortality data showed strong correlations with influenza A (r=0.65) and influenza B (r=0.50) infections [33].

Another study [46] explored the initial stages of implementing a simplified surveillance system in the Pacific Island countries and territories, with case definitions based solely on clinical signs and symptoms, eliminating the need for laboratory confirmation or detailed information on symptoms, location, sex, and age. ILI was defined as the sudden onset of fever, accompanied by a cough or sore throat. Countries, such as Fiji, Nauru, and Tuvalu, noted the usefulness of these simplified surveillance system definitions, with Fiji confirming ILI cases through laboratory tests [46].

Two distinct studies used ambulance dispatch data as part of their community-based syndromic surveillance [23,45]. One study [23] examined ambulance dispatch calls in New York City, United States, as a tool for monitoring ILI, and demonstrated that this syndromic surveillance method can successfully identify annual influenza epidemics, with 71 (3.2%) alarms triggered at the 99% confidence level. In comparison, another study [45] evaluated an influenza syndromic surveillance system in Santander, Spain, which relied on emergency data from prehospital emergency medical dispatch center call logs, ambulance service run-sheets, and ED patient records. The system achieved sensitivities of 70% and 63% and specificities of 89% and 95% for the 2010-11 and 2011-12 seasons, respectively [45].

#### Applications of Surveillance Systems Using Hospital and ED Data

Table 3 outlines 5 studies (17%) focusing on the use of surveillance systems in hospitals and EDs. The primary objective across these studies was the early detection of ARIs to mitigate disease spread and reduce health care burdens. One study [32] used hospitalization data to evaluate if a syndromic surveillance system could effectively detect local outbreaks of lower respiratory infections (LRIs). Space-time signals were generated, where if the observed number of cases in a specific space and time window exceeded the defined threshold, a warning signal was issued [32]. The study found that most signals indicating an LRI outbreak were related to influenza (60%) and RSV (70%) [32]. In contrast, another study [24] used data from ED visits in medical centers across California, Texas, and Florida to compare weekly rates of COVID-like symptoms (CLSs), ILIs, and non-ILIs during 5 flu seasons (2015-2020). The study focused particularly on the risk of illness during the early spread of SARS-CoV-2 during the 2019-2020 season [24]. Notably, while ED visits for ILIs and non-ILIs did not show substantial differences, the rates of CLSs were consistently lower in all seasons compared to the 2019-2020 season, with CLSs presenting at rates of –22%, –14%, and –20% for California, Texas, and Florida, respectively, relative to the average of the previous 4 seasons [24]. The study suggested that SARS-CoV-2 might have spread earlier than previously reported [24].

One study [35] evaluated trends for a surveillance system of severe acute respiratory infections (SARIs), by comparing different case definitions of SARIs based on the International Statistical Classification of Diseases and Related Health Problems 10th Revision (ICD-10), using hospital data from German hospitals. Three different SARI case definitions were applied: basic case definition, using only primary diagnoses; sensitive case definition, using primary and secondary diagnoses; and timely case definition, using only primary diagnoses of patients hospitalized for up to 1 week [35]. The results indicated that the sensitive case definition comprised 2.2 times as many patients as the basic case definition and 3.6 times as many as the timely case definition [35]. In comparison, another study [48] developed a forecasting model to predict the number of patients with influenza having ED visits due to fever. The correlation coefficient between the number of ED visits and the number of patients with influenza was the highest at day 7 (r=0.782; *P*<.001), and it remained above 0.70 (*P*=.001) up to the 14-day forecast interval, except on days 8, 9, and 12 [48].

A unique study [27] attempted to use a syndromic surveillance system to detect pertussis outbreaks in children under 10 years old by monitoring daily counts of ED visits with cough symptoms. The study showed that an increase of 10 notified cases of pertussis per day was associated with a 5.2% increase in ED visits with cough 7 days later (95% CI 1.005-1.100; *P*=.03) [27]. The median interval between the estimated onset of pertussis and case notification was 7 days [27].

**Table 3:** Summary of studies on surveillance systems using hospital and emergency department data.

Study	Study design	Country	Sample size (n)	Study population	Outcomes	Objectives	Findings	Limitations
Lukowsky et al [24], 2022	Retrospective	United States	California: n=520,026; Texas: n=392,444; Florida: n=467,628	Adults (veterans); Male gender: California: 466,656/520,026 (90%), Texas: 335,298/392,444 (85%), Florida: 412,739/467,628 (88%)	Effectiveness	Use data from ED^a^ visits at VA^b^ Medical Centers in California, Texas, and Florida to compare weekly rates of CLSs^c^, ILIs^d^, and non-ILIs during 5 consecutive flu seasons (2015-2020) to estimate the risk of developing each illness during the first 23 weeks of the 2019-2020 season.	Patients with CLSs were significantly more likely to visit the ED during the first 23 weeks of the 2019-2020 season compared to prior seasons, while ED visits for ILIs and non-ILIs did not differ substantially. Adjusted CLS risk was significantly lower for all seasons relative to the 2019-2020 season: RR15-16=0.72, 0.75, and 0.72; RR16-17=0.81, 0.77, and 0.79; RR17-18=0.80, 0.89, and 0.83; RR18-19=0.82, 0.96, and 0.81 in California, Texas, and Florida, respectively.	Examined ED visits among veterans who used VA centers. VA users are older than the US adult population, present with more chronic, physical, and mental conditions, are less educated, and have lower incomes, and about 90% are men. Given these limitations, the CLS rates in our study might be higher than among the general population.
Hong et al [48], 2022	Retrospective	South Korea	29,142,229	All ages	Accuracy	Use chief complaint data from the ED to detect the increment of influenza cases identified from nationwide medical service usage and develop a forecast model to predict the number of influenza patients accounting for the daily number of ED visits due to fever.	The correlation coefficient between the number of ED visits and the number of patients with influenza up to 14 days before the forecast, with the exceptions of days 8, 9, and 12, was higher than 0.70 (*P*=.001).	Identified patients with influenza were not confirmed cases based on laboratory diagnoses; Missing values in chief complaints; Lack of generalizability
Cashmore et al [27], 2013	Retrospective; Time series analysis	Australia	Pertussis notifications: n=12,311; Influenza notifications: n=4,332; Bronchiolitis ED visits: n=32,120	Under 10 years old	Features used and effectiveness	Daily counts of ED visits with cough, and whether the cough syndrome would respond to changes in the incidence of pertussis in children under the age of 10 years.	When notified pertussis increased by 10 cases in 1 day, ED visits with cough increased by 5.2% (RR 1.052, 95% CI 1.005-1.100; *P*=.03) 7 days later. Daily increases in the other independent variables had a smaller impact on cough visits. When notified influenza increased by 10 cases in 1 day, ED visits with cough increased by 0.8% (RR 1.008, 95% CI 1.000-1.017; *P*=.06) 7 days later.	Coding practices may vary among staff (information bias).
Buda et al [35], 2017	Retrospective (years 2012 to 2014); Prospective (years 2015 to 2016)	Germany	BCD^e^: n=63,168; SCD^f^: n=141,727; TCD^g^: n=39,787	Age: 0 to 65+ years	Features used and effectiveness	Compare the impact of different case definitions on the ability to capture SARI^h^ cases, to allow a timely trend analysis of the seasonal epidemic.	The SCD comprised 2.2 times as many patients as the BCD and 3.6 times as many as the TCD. The time course of SARI cases corresponded well with the results from primary care surveillance and influenza virus circulation. The patients fulfilling the TCD had been completely reported after 3 weeks, which was the fastest among case definitions.	Lack of complementary virological information
van den Wijngaard et al [32], 2010	Retrospective	Netherlands	1999-2004: n=222,638; 2005-2006: n=68,124	Age: 0 to 65+ years	Accuracy	Detect local outbreaks of lower-respiratory infections (LRIs) without swamping true signals by false alarms.	Recurrence interval ≥1 year: detected a total of 35 LRI clusters with 221 cluster signals; Recurrence interval ≥5 years: detected a total of 24 LRI clusters with 146 cluster signals (31% and 34% decreases, respectively). Most signals to determine an LRI outbreak were related to influenza (60%) and RSV^i^ (70%).	Prospective application of the system

^a^ED: emergency department.

^b^VA: Veterans Affairs.

^c^CLS: COVID-like symptom.

^d^ILI: influenza-like illness.

^e^BCD: basic case definition.

^f^SCD: sensitive case definition.

^g^TCD: timely case definition.

^h^SARI: severe acute respiratory infection.

^i^RSV: respiratory syncytial virus.

#### Applications of Surveillance Systems for Institutionalized Elderly People

Three studies (10%) examined the use of surveillance systems in institutions for elderly people (Table 4)*,* highlighting the critical role of EWSs in preparing for and managing respiratory infection outbreaks*.* Rosewell et al [25] assessed the impact of active surveillance on influenza outbreaks in aged-care facilities, reporting attack rates of 14% for residents and 5% for staff in the first outbreak, and 0% for residents and 6% for staff in the second outbreak. In contrast, a study conducted in Italy evaluated the preparedness of retirement and nursing homes during the initial spread of SARS-CoV-2 using questionnaires [28]. The study identified variability in staff training (53.8%), resident training (67.6%), availability of personal protective equipment (PPE) (41.7%), and infection control practices (73.5%) [28]. Finally, another study [26] explored the implementation of a novel web-based app, called Influenza Outbreak Communication, Advice and Reporting (FluCARE), to assist aged-care facilities in recognizing and managing influenza and COVID outbreaks in facilities. The app helped 60% of respondents in identifying the first few cases of ILIs, automatically notified 28% of respondents of a potential influenza outbreak, and helped 64% of respondents recognize if a facility had a COVID situation to monitor [26]. However, only 12% of respondents said that the app helped to identify the appropriate next steps for outbreak management.

**Table 4:** Summary of studies on surveillance systems for institutionalized elderly people.

Study	Study design	Country	Sample size	Study population	Outcomes	Objectives	Findings	Limitations
Rosewell et al [25], 2010	Clustered randomized trial	Australia	n=16 aged-care facilities; 377 residents	—^a^	Effectiveness	Evaluate the impact of active surveillance in influenza outbreaks at aged-care facilities.	Attack rate in the first outbreak: 14% (residents), 5% (staff), and 10% overall; Attack rate in the second outbreak: 0% (residents), 6% (staff), and 3% overall.	Generalizability; Lack of routine measurements in some cases
Gugliotta et al [28], 2021	Cross-sectional (observational)	Italy	n=14 facilities	—	Effectiveness	Evaluate the perceived risk by all residential facilities for elderly people following the first wave of the SARS-CoV-2 epidemic to identify critical control points in order to provide an intervention model that could be used for a large-scale preparedness assessment of these facilities.	Application of good practices (median values): restriction policies (87.5%), staff training (53.8%), resident training (67.6%), availability of personal protective equipment (41.7%), infection control practices (73.5%), and communication (80%).	Confounding factors; Generalizability
Quinn et al [26], 2023	Prospective cohort (mixed methods)	Australia	n=31	—	Accuracy	Early detection and response to influenza and COVID-19 outbreaks in aged-care facilities through the use of the FluCARE app.	FluCARE helped 15/25 (60%) respondents in identifying the first few cases of influenza-like illnesses. The app automatically notified 7/25 (28%) respondents of a potential influenza outbreak occurring within the facility. The app helped 16/25 (64%) respondents in recognizing if a facility had a COVID-19 situation to monitor. Only 2 of 25 (12%) respondents said that the app helped to identify the appropriate next steps to manage and control outbreaks within facilities.	Selection bias

^a^Not applicable.

#### Applications of Machine Learning in the Development of EWSs

Table 5 summarizes 4 studies (14%) that used machine learning (ML) algorithms to enhance EWSs for timely detection and management of COVID. All 4 studies explored novel ML approaches to assist in the early detection of infection by identifying common factors leading to severe COVID-19 infection.

**Table 5:** Summary of studies applying machine learning models in the development of early warning systems.

Study	Study design	Country	Sample size	Study population	Outcomes	Objectives	Findings	Limitations
Jakob et al [34], 2022	Cross-sectional	Germany	n=1223 (model discovery); n=2264 (validation)	—^a^	Features used and accuracy	Implement a machine learning-based predictor and score for patients with asymptomatic or mild COVID-19 at risk of progressing to advanced COVID-19.	Predictor model: AUC^b^=0.77 (SD 0.02); OR^c^=6.78, 95% CI 2.74-16.65; Score (SACOV-19): AUC=0.73 (SD 0.01)	Generalizability; Overrepresentation of co-morbidities; Missing values for variables
Chang et al [30], 2022	Cross-sectional	China	1059	—	Features used, effectiveness, and accuracy	Conduct a logistic regression analysis for factors related to severe versus common-type COVID-19, and establish an early warning nomogram model for severe COVID-19 to guide early and timely treatment.	Logistic regression model: AUC=0.863, with a sensitivity of 72.1% and specificity of 86.4% (95% CI 0.836-0.889; *P*<.001); Accuracy of the nomogram: AUC=0.889, with a sensitivity of 93.9% and specificity of 87.8% (95% CI 0.828-0.950; *P*<.001).	Small sample size; Generalizability
El Halabi et al [21], 2022	Cross-sectional	United States	5859	Age: 18+ years	Features used and accuracy	Understand the determinants of the need for hospitalization and the projected LOS^d^ by constructing a web-based predictive model using logistic regression.	AUC of 0.881 (95% CI 0.872-0.890) for hospitalization and 0.770 (95% CI 0.752-0.789) for LOS. Elevated levels of CRP, creatinine, and ferritin were key determinants of hospitalization and LOS (*P*<.05).	Generalizability
Alavi et al [20], 2022	Prospective	United States	3318, with wearable data available for 2155	—	Accuracy	Create a real-time monitoring and alerting system for detecting abnormal physiological events, including COVID-19 infection onset, using agnostic algorithms across different smartwatches.	Among 3318 participants, 278 reported a positive COVID-19 test, but 84 had sufficient wearable data around the time of infection. The system generated presymptomatic and asymptomatic alerts for COVID-19 in 67 (80%) of infected individuals, with presymptomatic signals being observed about 3 days (median) before symptom onset.	Missing data

^a^N/A: not applicable.

^b^AUC: area under the curve.

^c^OR: odds ratio.

^d^LOS: length of stay.

Jakob et al [34] developed the SACOV-19 score using ML to assess the risk of patients with asymptomatic or mild COVID-19 progressing to advanced stages. The score was derived from a minimalistic predictor system based on 20 out of 473 patient variables and demonstrated good performance, with an area under the curve (AUC) of 0.77 (SD 0.02) and an odds ratio (OR) of 6.78 (95% CI 2.74-16.65) [34]. Similarly, Chang et al [30] created an early warning nomogram using logistic regression to identify factors leading to severe COVID. The model, which enrolled 1059 COVID patients, achieved an AUC of 0.863, with a sensitivity of 72.1% and specificity of 86.4% (95% CI 0.836-0.889; *P*<.001) [30]. The accuracy of the nomogram for disease progression was validated in a cohort of 123 patients, with an AUC of 0.889, sensitivity of 93.9%, and specificity of 87.8% (95% CI 0.828-0.950; *P*<.001) [30].

Likewise, another study employed logistic regression to develop predictive models for hospitalization and length of stay (LOS) in COVID patients presenting to the ED [21]. The web-based predictive model identified several key factors influencing hospitalization and LOS, achieving AUCs of 0.881 (95% CI 0.872-0.890) for hospitalizations and 0.770 (95% CI 0.752-0.789) for LOS [21]. Comparatively, Alavi et al [20] used agnostic algorithms to create a real-time monitoring and alerting system using wearable devices such as smartwatches for detecting COVID infection. Among 3318 participants, 278 reported a positive COVID-19 test, but only 84 had sufficient wearable data around the time of COVID infection [20]. The system generated presymptomatic and asymptomatic alerts for COVID in 67 (80%) infected individuals, with presymptomatic signals being observed about 3 days (median) before symptom onset [20].

#### Applications of Previously Implemented Surveillance Systems

Table 6 presents 2 studies (7%) that assessed the adaptation of existing EWSs in a hospital setting during the COVID pandemic. Both studies highlighted the importance of re-establishing these systems for health crises, such as the SARS-CoV-2 pandemic, emphasizing the need for critical factors such as effective management and PPE. A study by Yohannes et al [22] explored the implementation of the COVID early warning system (CEWS) protocol, which aimed to reduce the risk of intensive care unit (ICU) admission in patients with COVID-19, while ensuring effective management on medical floors. The study enrolled 1024 inpatients, with those in the CEWS intervention group demonstrating a lower likelihood of ICU admission (hazard ratio [HR] 0.73, 95% CI 0.53-1.000; *P*=.0499) and shorter ICU stay if admitted (HR for ICU discharge: 1.74, 95% CI 1.21-2.51; *P*=.003) [22]. Comparably, a study by Htun et al [41] assessed the implementation of the staff health surveillance system (S3) at Tan Tock Seng Hospital (TTSH) in Singapore, which was initially implemented during the 2003 SARS outbreak. The system aimed to protect health care workers from nosocomial SARS-CoV-2 infection through PPE, staff fever and sickness surveillance, and enhanced medical surveillance [41]. Among 1524 frontline staff under surveillance, there was a median of 8 staff illness episodes per day, with 10% (n=29) resulting in hospitalizations, although none tested positive for SARS-CoV-2 [41].

**Table 6:** Summary of studies on previously implemented surveillance systems.

Study	Study design	Country	Sample size	Study population	Outcomes	Objectives	Findings	Limitations
Yohannes et al [22], 2023	Retrospective cohort	United States	1024	Age: 18+ years	Effectiveness	Evaluate the impact of the COVID EWS^a^ (CEWS) protocol on ICU^b^ capacity while determining the safety of keeping patients with severe COVID-19 on medical floors under augmented monitoring.	CEWS intervention: Less likely to be admitted to the ICU (HR^c^ 0.73, 95% CI 0.53-1.000; *P*=.0499). Patients admitted from medical floors to the ICU had shorter ICU stays (HR for ICU discharge: 1.74, 95% CI 1.21-2.51; *P*=.003). No significant difference between the CEWS intervention and control in the need for mechanical ventilation (OR^d^ 0.93, 95% CI 0.38-2.31; *P*=.88) or mortality (OR 0.79, 95% CI 0.52-1.18; *P*=.25).	Generalizability; Retrospective study; Missing data for a few points in the SOFA^e^ score
Htun et al [41], 2020	Cross-sectional	Singapore	10,583	—^f^	Features used and effectiveness	Evaluate the protection and safety of health care workers regarding getting COVID-19 through the implementation of personal protective equipment and staff sickness surveillance systems.	Median of 8 staff illness episodes per day; total of 287 illness episodes within the study period, with 10% (n=29) resulting in hospitalizations.	Self-reporting; hence, the accuracy of illness prediction can be questioned.

^a^EWS: early warning system.

^b^ICU: intensive care unit.

^c^HR: hazard ratio.

^d^OR: odds ratio.

^e^SOFA: sepsis-related organ failure assessment.

^f^Not applicable.

#### Applications of the NEWS

Three studies (10%) explored the implementation or comparison of the NEWS in the context of COVID (Table 7). The findings indicated that while the NEWS was effective, modifications to the surveillance system specifically for COVID-19 management outperformed the initial approach. Khuraijam et al [39] examined the effectiveness of NEWS 2 at triage in the ED for identifying patients with COVID at risk of critical illness, clinical deterioration, or hospital mortality within 24 hours of admission, compared to the quick sepsis-related organ failure assessment (qSOFA) score. Both NEWS 2 (area under the receiver operating characteristic curve [AUROC]=0.883) and qSOFA (AUROC=0.851) effectively identified patients at risk, with no significant difference in diagnostic performance between the 2 scores (*P*=.31) [39]. Similarly, another study developed and validated a COVID-19 early warning score (COEWS) and compared it with NEWS 2 [43]. COEWS was calculated from widely available and affordable laboratory parameters, such as complete blood count and oxygen saturation values, and showed similar AUROC values in vaccinated (0.743) and unvaccinated (0.767) patients in the external validation cohort, outperforming NEWS 2 (AUROC 0.677 in vaccinated patients and 0.648 in unvaccinated patients) [43]. Comparatively, Tagliabue et al [29] used a modified version of NEWS specifically for COVID-19 management (m-NEWS). The study aimed at comparing the triage performance of m-NEWS at admission with respect to the age variable alone for the recovery of COVID patients. The study found that m-NEWS at admission (AUROC=0.813) outperformed age (AUROC=0.747) as a classifier for patient outcomes [29].

**Table 7:** Summary of studies on the application of the National Early Warning Score (NEWS).

Study	Study design	Country	Sample size	Study population	Outcomes	Objectives	Findings	Limitations
Khuraijam et al [39], 2022	Retrospective cohort	India	104 patients	Age: 18+ years	Effectiveness	Determine if the application of the NEWS^a^ 2 score at triage in the ED^b^ with COVID-19 patients was more effective in identifying patients with critical illness, clinical deterioration, or hospital mortality within 24 hours of admission, compared to the qSOFA^c^ score.	NEWS 2: 25% patients required continuous monitoring, of which 12.7% subsequently deteriorated within 24 hours of admission and 7% died. NEWS 2 (threshold 0;1, AUROC^d^ 0.883, 95% CI 0.8-0.966) and qSOFA (threshold 0;1, AUROC 0.851, 95% CI 0.766-0.936) effectively identified patients in the ED at risk for clinical deterioration. No significant difference in the diagnostic performance between qSOFA and NEWS 2 (*P*=.31).	Limited availability of PCR^e^ tests; Small sample size
Klén et al [43], 2023	Retrospective cohort	Spain and Argentina	15,903 hospital admissions	—^f^	Features used and effectiveness	Develop and implement COEWS^g^, an EWS^h^ that was automatically calculated from available and affordable laboratory parameters for patients with COVID-19.	AUROC=0.700 (95% CI 0.654-0.745) for the COEWS performed with the scores. AUROC was 0.743 (0.703-0.784) in vaccinated patients and 0.767 (0.749-0.785) in unvaccinated patients when the COEWS was assessed using the external validation cohort. NEWS 2 presented an AUROC of 0.677 (95% CI 0.601-0.752) in vaccinated patients and 0.648 (95% CI 0.608-0.689) in unvaccinated patients.	Missingness in some parameters, resulting in selection bias; Generalizability
Tagliabue et al [29], 2021	Retrospective	Italy	225 patients	Average age: 71 years	Features used and accuracy	Identify any correlation between m-NEWS^i^ on admission and clinical outcomes.	The visible distance between the curves, along with AUC^j^ values, indicated that in the statistical sample, the m-NEWS at admission (AUC=0.813) vastly outperformed age (0.747) as a classifier for the “recovery” outcome.	Generalizability

^a^NEWS: National Early Warning Score.

^b^ED: emergency department.

^c^qSOFA: quick sepsis-related organ failure assessment.

^d^AUROC: area under the receiver operating characteristic curve.

^e^PCR: polymerase chain reaction.

^f^Not applicable.

^g^COEWS: COVID-19 early warning score.

^h^EWS: early warning score.

^i^m-NEWS: modified National Early Warning Score specifically for COVID-19 management.

^j^AUC: area under the curve.

### Quality Assessments

Our quality assessment analysis revealed that the majority of studies achieved a score of 6 (11/29, 38%) [20,21,27,29-31,34,35,38,39,48] or 5 (9/29, 31%) [22,26,32,36,40,42,43,45,47], indicating that these studies were of high value, relevance, and reliability, with clearly defined research objectives and thoroughly analyzed results. However, a few studies scored 4 (7/29, 24%) [23-25,28,33,44,46] or 3 (2/29, 7%) [37,41], indicating some inconsistencies in the findings. These lower scores were often due to studies not comparing their models to existing surveillance systems, using datasets that lacked reproducibility, and presenting results that were not thoroughly analyzed, which could affect their overall reliability. A detailed breakdown of each study’s assessment is presented in Table 8.

**Table 8:** Quality assessment results using the Critical Appraisal Skills Programme (CASP) tool.

Author and year	Is there a clear statement of the aim of the research?^a^	Is the test dataset reproducible?^a^	Is it clearly stated in the study which other algorithms the study’s algorithms have been compared with?^a^	Was the methodology of evaluation and/or the early warning system (EWS) functioning process described?^a^	Are the test results thoroughly analyzed? (EWS sensitivity/specificity, EWS effectiveness as an early predictor, and pros and cons of the system)^a^	Did the study have limitations or risk of bias mentioned by the authors?^a^	Total score
Jakob et al [34], 2022	1	1	1	1	1	1	6
Chang et al [30], 2022	1	1	1	1	1	1	6
Htun et al [41], 2020	1	1	0	0	0	1	3
Yohannes et al [22], 2023	1	1	1	1	0	1	5
Khuraijam et al [39], 2022	1	1	1	1	1	1	6
Klén et al [43], 2023	1	1	1	1	0	1	5
Tagliabue et al [29], 2021	1	1	1	1	1	1	6
Rosewell et al [25], 2010	1	0	1	1	0	1	4
Gugliotta et al [28], 2021	1	1	0	1	0	1	4
Quinn et al [26], 2023	1	1	0	1	1	1	5
El Halabi et al [21], 2022	1	1	1	1	1	1	6
Seck et al [40], 2023	1	1	1	1	0	1	5
Lee et al [38], 2021	1	1	1	1	1	1	6
Ferreira et al [42], 2022	1	1	1	0	1	1	5
Awekeya et al [37], 2021	1	0	0	1	0	1	3
Alavi et al [20], 2022	1	1	1	1	1	1	6
Katayama et al [47], 2020	1	0	1	1	1	1	5
Kavanagh et al [44], 2012	1	0	1	1	0	1	4
Dong et al [31], 2016	1	1	1	1	1	1	6
Nuvey et al [36], 2019	1	0	1	1	1	1	5
Mostashari et al [23], 2003	1	1	0	1	0	1	4
Schrell et al [45], 2013	1	0	1	1	1	1	5
van den Wijngaard et al [33], 2008	1	0	1	1	0	1	4
van den Wijngaard et al [32], 2010	1	1	1	1	0	1	5
Cashmore et al [27], 2013	1	1	1	1	1	1	6
Hong et al [48], 2022	1	1	1	1	1	1	6
Lukowsky et al [24], 2022	1	0	1	1	0	1	4
Buda et al [35], 2017	1	1	1	1	1	1	6
Kool et al [46], 2012	1	0	1	1	0	1	4

^a^1=yes; 0=no.

## Discussion

### Principal Findings

The findings from our scoping review demonstrate the varied and critical roles that EWSs play in detecting and managing acute respiratory illnesses across diverse settings. We identified 29 studies that explored the application of EWSs in several contexts, including community-based settings, hospitals, EDs, care facilities for elderly people, previously implemented surveillance systems, development of EWSs using ML and algorithms, and applications of the national EWS. These studies collectively highlight the advantages of EWSs in improving response times, enhancing health outcomes, and offering potential applications across different health care settings. However, the review highlights significant limitations, including challenges in generalizability and data reliability, and the need for robust validation methods.

A key finding across the studies included in this review is the crucial role of EWSs in reducing response times and improving health outcomes. This aligns with previous reviews that highlighted the proactive capabilities of EWSs in detecting outbreaks through the compilation of prediagnostic data [7]. However, a prior systematic review cautioned that while EWSs compiling prediagnostic data are effective in early outbreak detection, they should not be considered as the sole reliable indicator for outbreak detection [7]. This is due to insufficient research on the implementation and feasibility of EWSs in low- and middle-income countries, as well as in settings with limited resources [7]. The systematic review also revealed that in deprived areas, staff training was essential to the implementation of electronic EWSs. Our review reached a similar conclusion, emphasizing that even in high-income regions and countries, staff training and adherence were crucial for the successful implementation and appropriate use of EWSs [25,26,28]. Both our study and previous reviews highlighted the importance of staff training and adherence to successfully implement and appropriately use EWSs, even in high-income regions. This underscores the need for further large-scale trials to standardize the use of EWSs for detecting infectious disease outbreaks.

Our review also explored the advantages and limitations of various EWS applications. Community-based EWSs, for instance, demonstrated versatility and utility, particularly in leveraging self-reported data for surveillance. Studies like those by Seck et al [40] assessed surveillance systems, where community members reported suspected COVID cases. Similarly, studies by Kavanagh et al [44] and Katayama et al [47] demonstrated the potential efficacy of telephone-based surveillance systems in detecting influenza within communities. In another study, various sources of syndromic surveillance were investigated, noting that internet searches for “fever” strongly correlated with influenza activity, and school absence reporting detected influenza activity a week earlier than laboratory confirmation [31]. Self-reported data were also collected in the FluWatchers program implemented in Canada, where community members voluntarily provided information regarding ILI symptoms [38]. Additionally, 2 studies analyzed the use of community institutions and facilities as vectors for COVID testing and reporting by community members, further emphasizing the versatility and utility of community-based approaches in surveillance [37,42]. These examples underscore the challenges and the crucial role of reliability and validity in self- and community-reported data, illustrating the complex dynamics of implementing community-based EWSs effectively.

Hospital-based surveillance systems have been underscored as a valuable complement to community-based outbreak detection or laboratory surveillance [32,33]. These systems, which used multiple data sources, such as pathogen counts and syndromic data, can identify distinct syndrome elevations that cannot be explained by a single source, such as routine laboratory pathogen tests [33]. The potential of hospital data to supplement surveillance systems has also been noted, with studies exploring the early spread of SARS-CoV-2 within communities [24]. Furthermore, the value of ambulance dispatch data, in addition to ED data, has been highlighted in reflecting community health trends, which can inform broader surveillance strategies [23,45]. The integration of diverse data sources (including data from hospitals and EDs as well as community and laboratory surveillance data) significantly enhances the robustness of syndromic surveillance systems. This comprehensive approach facilitates more effective disease detection and management strategies by providing a comprehensive view of health trends across various settings. By amalgamating these distinct data streams, surveillance systems can achieve a more nuanced understanding of epidemiological patterns, which ultimately enables timely and targeted public health interventions.

In aged-care facilities, where elderly people are at a high risk of severe complications from acute respiratory infectious diseases, such as COVID, the implementation of EWSs is especially critical. Studies by Rosewell et al [25], Gugliotta et al [28], and Quinn et al [26] have explored how EWSs can monitor and manage outbreaks effectively in these settings. They highlight the crucial role of staff training and the availability of adequate resources, such as PPE, to ensure the operational effectiveness of EWSs. They further emphasize that the active involvement of staff in surveillance is an essential step for the timely detection and management of outbreaks. This highlights the need for a comprehensive approach to implementing EWSs in aged-care facilities to safeguard the health of vulnerable populations.

The role of ML in EWSs is becoming increasingly pivotal. EWSs often employ vital signs to detect patient deterioration [49], and ML models, when integrated into electronic health records, can analyze extensive datasets to identify patterns indicating potential health declines [30,34]. As evidenced by studies present in this review, EWSs that have been designed to detect patterns of abnormality in patient variables, such as body temperature or respiration rate, can effectively track the progression of patient outcomes [30,34]. Further studies have found that using ML models, including logistic regression, tree-based methods, kernel-based methods, and neural networks, can accurately predict the risk of deterioration in patients [49]. This emphasizes the significant role of ML in enhancing the predictive accuracy and effectiveness of EWSs.

Several studies have used, modified, or compared existing EWSs for the detection of acute respiratory illnesses. NEWS, and its successor, NEWS 2, are standardized clinical scoring systems developed to improve the detection of deterioration in acutely ill patients. In these studies, alternative EWSs, such as qSOFA and COEWS, were employed and compared with NEWS 2 [39,43]. One study found no significant difference between qSOFA and NEWS 2 in detecting patient deterioration [39]. Conversely, another study demonstrated that COEWS, which incorporates additional clinical parameters like complete blood count, blood glucose, and oxygen saturation, yielded superior results in patient outcome detection compared to NEWS 2 [43]. Additionally, a study used a modified version of NEWS (m-NEWS), which highlighted age as a significant factor in assessing patient outcomes [29]. Furthermore, other research has focused on previously implemented surveillance systems that were modified or adapted for COVID outbreaks. These studies involved surveillance systems, such as CEWS and S3, which monitored the vital signs of patients and staff to mitigate ICU admissions and reduce episodes of staff illness [22,41]. This approach underscores the adaptability of EWSs in responding to emergent health threats by integrating comprehensive clinical data to guide early intervention.

The reviewed studies on EWSs for COVID and other health threats commonly faced several limitations that impacted the reliability and generalizability of their findings. A major challenge was the limited generalizability of results across different settings and populations [21,22,24-26,34,37,38,40-42]. Many studies focused on EWSs in specific environments, such as hospitals [24,32,35,48], regions [21,23,27,31,36,37,42,45,47], or countries [30,32-34,38,43,44,46], but the applicability of these findings to other public health jurisdictions remains uncertain. Furthermore, some studies relied on small sample sizes for validating or testing EWSs, which could diminish the statistical power and robustness of the results [30,36,39]. Another limitation is the potential bias introduced by self-reported symptoms [26,40,41], which may not accurately reflect the true prevalence or severity of the disease. This bias could also be influenced by the willingness and acceptance of users to adopt new technologies. Moreover, the quality of the EWS model analysis may be compromised by incomplete or delayed reporting and testing, leading to possible underdetection or delayed notification of cases by health care workers or authorities [20,22,27,40,41,47]. Additionally, only 1 study specifically targeted pediatric populations [27], with most studies focusing on all age groups [23,31,33,36,44-48]. These common limitations highlight the need for studies with larger more diverse study populations, improved data collection methods, and further validation to enhance the reliability and generalizability of EWS findings.

Moving forward, future research should focus on standardizing EWSs across different health care settings, ensuring their applicability in low-resource environments and integrating artificial intelligence and particularly ML to improve predictive capabilities. Furthermore, international collaboration will be crucial in addressing these challenges, as it can facilitate the sharing of best practices, data, and resources to develop more effective and universally applicable EWSs.

The strengths of this review include a comprehensive search across multiple databases, along with establishing clearly defined inclusion and exclusion criteria. The structured study selection process added robustness to the review. Additionally, employing a modified CASP tool to assess the quality of the studies enhanced the credibility of our findings. However, this review has limitations that need to be acknowledged. The variability in the quality of the included studies, as reflected by the CASP scores, suggests potential inconsistencies in the study findings.

### Conclusion

This scoping review has systematically examined the literature on the deployment and efficacy of EWSs across a variety of settings for the detection of acute respiratory illnesses. While the studies demonstrated the potential of EWSs in enhancing disease surveillance and response capabilities, significant barriers remain. The primary challenges identified include the limited generalizability of EWSs across different geographical and clinical settings, the variability in data quality and reporting standards, and the need for more robust validation methods to ensure system reliability and effectiveness. Furthermore, while some EWSs employ artificial intelligence, the integration of these tools into existing public health infrastructure requires careful consideration of ethical, logistical, and technical factors. As EWSs continue to evolve, there is a critical need for ongoing research and development. Future studies should focus on standardizing EWS protocols, expanding their applicability to low-resource settings, and enhancing their ability to process and analyze real-time data. Additionally, it is imperative to involve stakeholders in the design and implementation of these systems to ensure they meet the diverse needs of various populations. Ultimately, EWSs have the potential to significantly reduce the impact of respiratory outbreaks on health care systems by enabling earlier detection and more coordinated response efforts. However, the complete realization of this potential requires a concerted effort to address the current limitations and ensure that these systems are as effective and inclusive as possible.

## Appendix

Multimedia Appendix 1 PRISMA-ScR (Preferred Reporting Items for Systematic Reviews and Meta-Analyses extension for Scoping Reviews) checklist.

## Abbreviations

ARI: acute respiratory infection
AUC: area under the curve
AUROC: area under the receiver operating characteristic curve
CASP: Critical Appraisal Skills Programme
CEWS: COVID early warning system
CLS: COVID-like symptom
COEWS: COVID-19 early warning score
ED: emergency department
EWS: early warning system
HR: hazard ratio
ICU: intensive care unit
ILI: influenza-like illness
LOS: length of stay
LRI: lower respiratory infection
ML: machine learning
NEWS: National Early Warning Score
PPE: personal protective equipment
PVP: predictive value positive
qSOFA: quick sepsis-related organ failure assessment
RSV: respiratory syncytial virus
SARI: severe acute respiratory infection

## Data Availability

The datasets used or analyzed during this study are available from the corresponding author upon reasonable request.
