# Supplementary material for: Early Warning Systems for Acute Respiratory Infections: Scoping Review of Global Evidence
Source: JMIR Public Health Surveill. 2024 Nov 7;10:e62641. doi: 10.2196/62641 (PMC11582482; doi:10.2196/62641)
Supplement: Multimedia Appendix 1 [file publichealth_v10i1e62641_app1.docx]

**Preferred Reporting Items for Systematic reviews and Meta-Analyses extension for Scoping Reviews (PRISMA-ScR) Checklist**

| **SECTION** | **ITEM** | **PRISMA-ScR CHECKLIST ITEM** | **REPORTED ON PAGE #** |
| --- | --- | --- | --- |
| **TITLE** | | | |
| Title | 1 | Identify the report as a scoping review. | Page 1 |
| **ABSTRACT** | | | |
| Structured summary | 2 | Provide a structured summary that includes (as applicable): background, objectives, eligibility criteria, sources of evidence, charting methods, results, and conclusions that relate to the review questions and objectives. | Page 2 |
| **INTRODUCTION** | | | |
| Rationale | 3 | Describe the rationale for the review in the context of what is already known. Explain why the review questions/objectives lend themselves to a scoping review approach. | Pages 4 |
| Objectives | 4 | Provide an explicit statement of the questions and objectives being addressed with reference to their key elements (e.g., population or participants, concepts, and context) or other relevant key elements used to conceptualize the review questions and/or objectives. | Pages 4 |
| **METHODS** | | | |
| Protocol and registration | 5 | Indicate whether a review protocol exists; state if and where it can be accessed (e.g., a Web address); and if available, provide registration information, including the registration number. | Page 4 |
| Eligibility criteria | 6 | Specify characteristics of the sources of evidence used as eligibility criteria (e.g., years considered, language, and publication status), and provide a rationale. | Pages 4-5 |
| Information sources* | 7 | Describe all information sources in the search (e.g., databases with dates of coverage and contact with authors to identify additional sources), as well as the date the most recent search was executed. | Pages 4-5 |
| Search | 8 | Present the full electronic search strategy for at least 1 database, including any limits used, such that it could be repeated. | Sample Included below: Appendix 1 |
| Selection of sources of evidence† | 9 | State the process for selecting sources of evidence (i.e., screening and eligibility) included in the scoping review. | Pages 4-5 |
| Data charting process‡ | 10 | Describe the methods of charting data from the included sources of evidence (e.g., calibrated forms or forms that have been tested by the team before their use, and whether data charting was done independently or in duplicate) and any processes for obtaining and confirming data from investigators. | Page 5 |
| Data items | 11 | List and define all variables for which data were sought and any assumptions and simplifications made. | Page 5 |
| Critical appraisal of individual sources of evidence§ | 12 | If done, provide a rationale for conducting a critical appraisal of included sources of evidence; describe the methods used and how this information was used in any data synthesis (if appropriate). | Page 5 |
| Synthesis of results | 13 | Describe the methods of handling and summarizing the data that were charted. | Page 5 |
| **RESULTS** | | | |
| Selection of sources of evidence | 14 | Give numbers of sources of evidence screened, assessed for eligibility, and included in the review, with reasons for exclusions at each stage, ideally using a flow diagram. | Page 6 (and Figure 1) |
| Characteristics of sources of evidence | 15 | For each source of evidence, present characteristics for which data were charted and provide the citations. | Page 6 and Tables 1-6 |
| Critical appraisal within sources of evidence | 16 | If done, present data on critical appraisal of included sources of evidence (see item 12). | Pages 32-35 and Table 7 |
| Results of individual sources of evidence | 17 | For each included source of evidence, present the relevant data that were charted that relate to the review questions and objectives. | Tables 1 - 7 |
| Synthesis of results | 18 | Summarize and/or present the charting results as they relate to the review questions and objectives. | Pages 5-35 |
| **DISCUSSION** | | | |
| Summary of evidence | 19 | Summarize the main results (including an overview of concepts, themes, and types of evidence available), link to the review questions and objectives, and consider the relevance to key groups. | Pages 35-37 |
| Limitations | 20 | Discuss the limitations of the scoping review process. | Pages 37 |
| Conclusions | 21 | Provide a general interpretation of the results with respect to the review questions and objectives, as well as potential implications and/or next steps. | Pages 38 |
| **FUNDING** | | | |
| Funding | 22 | Describe sources of funding for the included sources of evidence, as well as sources of funding for the scoping review. Describe the role of the funders of the scoping review. | Page 1 |

JBI = Joanna Briggs Institute; PRISMA-ScR = Preferred Reporting Items for Systematic reviews and Meta-Analyses extension for Scoping Reviews.

* Where *sources of evidence* (see second footnote) are compiled from, such as bibliographic databases, social media platforms, and Web sites.

† A more inclusive/heterogeneous term used to account for the different types of evidence or data sources (e.g., quantitative and/or qualitative research, expert opinion, and policy documents) that may be eligible in a scoping review as opposed to only studies. This is not to be confused with *information sources* (see first footnote).

‡ The frameworks by Arksey and O’Malley (6) and Levac and colleagues (7) and the JBI guidance (4, 5) refer to the process of data extraction in a scoping review as data charting*.*

§ The process of systematically examining research evidence to assess its validity, results, and relevance before using it to inform a decision. This term is used for items 12 and 19 instead of "risk of bias" (which is more applicable to systematic reviews of interventions) to include and acknowledge the various sources of evidence that may be used in a scoping review (e.g., quantitative and/or qualitative research, expert opinion, and policy document).

*From:* Tricco AC, Lillie E, Zarin W, O'Brien KK, Colquhoun H, Levac D, et al. PRISMA Extension for Scoping Reviews (PRISMAScR): Checklist and Explanation. Ann Intern Med. 2018;169:467–473. [doi: 10.7326/M18-0850](http://annals.org/aim/fullarticle/2700389/prisma-extension-scoping-reviews-prisma-scr-checklist-explanation).

**Appendix 1: MEDLINE Search Strategy**

Search for: 36 or 37
Results: 100
Database: Ovid MEDLINE(R) and Epub Ahead of Print, In-Process, In-Data-Review & Other Non-Indexed Citations

1     "early adj1 warning adj1 methods".ab,ti. (0)
2     ("early adj1 warning adj1 system*" or "early adj1 warning adj1 protocol*").ab,ti. (0)
3     "early warning".ab,ti. (10183)
4     screen*.tw. (976351)
5     surveillance.ab,ti. (233531)
6     exp Immunologic Surveillance/ (1984)
7     mass screening/ (116512)
8     (early adj1 detection).ab,ti. (82022)
9     forecasting/ (91747)
10     forecast*.ab,ti. (28216)
11     diagnosis/ or clinical decision-making/ or clinical reasoning/ or diagnosis, computer-assisted/ or image
interpretation, computer-assisted/ or diagnosis, differential/ or "diagnostic techniques and procedures"/ or early
diagnosis/ or exp prognosis/ (2469942)
12     predict*.tw. (2068828)
13     "early adj2 diagnos*".tw. (0)
14     or/1-13 (5317704)
15     Respiratory tract infections/ or exp bronchitis/ or exp common cold;/ or exp influenza, human/ or laryngitis/ or
exp paryngitis/ or exp pleurisy/ or exp pneumonia/ or exp rhinitis/ or exp rhinoscleroma/ or expo severe acute
respiratory syndrome/ or exp sinusitis/ or exp supraglottis/ or exp tracheitis/ or exp whooping cough/ (526562)
16     coronaviridae infections/ or coronavirus infections/ or SARS Virus/ (50123)
17     (coronavirus* or "corona virus*" or mers or "middle east respiratory syndrome*" or "Severe Acute Respiratory
Syndrome*" or SARS or CoV or SARS-CoV or MERS-CoV or 2019-nCoV or COVID-19 or "2019 novel coronavirus disease" or "2019
ncov disease" or "2019 ncov infection" or "coronavirus disease 19" or "severe acute respiratory syndrome coronavirus 2"
or "severe acute respiratory syndrome coronavirus 2" or "wuhan" or "sars cov 2").tw,kf. (400029)
18     (flu or influenza or "respiratory tract infection*" or "respiratory infection*" or bronchitis or "common cold" or
laryngitis or pharyngitis or pneumonia or rhinitis or rhinoscleroma or sinusitis or supraglottitis or tracheitis or
"whooping cough").tw,kf. (397166)
19     or/15-18 (880904)
20     implementation science/ (1310)
21     exp Program Evaluation/ (84662)
22     (implement or implementation or implements or implementing).ab,ti. (509508)
23     exp guideline/ (37915)
24     protocol.ab,ti. (422446)
25     clinical decision rules/ (930)
26     principle.ab,ti. (125270)
27     or/20-26 (1126809)
28     14 and 19 and 27 (11536)
29     14 and 27 (255914)
30     limit 29 to covid-19 (6885)
31     28 or 30 (11556)
32     "cross-sectional:".tw. (528370)
33     "in vivo".tw. (1065770)
34     cohort.tw. (795499)
35     or/32-34 (2339190)
36     limit 31 to randomized controlled trial (676)
37     31 and 35 (1569)
38     36 or 37 (2209)

***************************
References

Abbasian, S., Razmi, M., Bahramian, H., Shanbehzadeh, M., Kazemi-Arpanahi, H. (2023). Diagnosis and treatment of
coagulopathy caused by the new coronavirus: a systematic review and meta-analysis protocol..  Advanced Biomedical
Research, , 12, 147. <https://dx.doi.org/10.4103/abr.abr_403_21>
 
Link to the Ovid Full Text or citation: <https://ovidsp.ovid.com/ovidweb.cgi?T=JS&CSC=Y&NEWS=N&PAGE=fulltext&D=pmnm&AN=37564459>
 

Abuauf, M., Raboe, E. H., Alshareef, M., Rabie, N., Zailaie, R., Alharbi, A., Felemban, W., Alnasser, I., Shalaby, H.
(2023). Covid-19 conceptual modeling: single-center cross-sectional study..  JMIR Formative Research, , 7, e41376.
<https://dx.doi.org/10.2196/41376>
 
Link to the Ovid Full Text or citation: <https://ovidsp.ovid.com/ovidweb.cgi?T=JS&CSC=Y&NEWS=N&PAGE=fulltext&D=pmnm&AN=37256829>
 

Ahmad, R. A., Indriani, C., Arisanti, R. R., Nanda, R. O., Mahendradhata, Y., Wibawa, T. (2023). Seroprevalence of
sars-cov-2 and risk factors in bantul regency in march-april 2021, yogyakarta, indonesia..  PLOS Global Public Health, ,
3(6), e0000698. <https://dx.doi.org/10.1371/journal.pgph.0000698>
 
Link to the Ovid Full Text or citation: <https://ovidsp.ovid.com/ovidweb.cgi?T=JS&CSC=Y&NEWS=N&PAGE=fulltext&D=pmnm&AN=37363894>
 

Alaa, A., Sarhan, N., Lotfy El-Ansary, M. G., Bazan, N. S., Farouk, K., Ismail, R. S., Schalaan, M. F., Abd-Allah, A. R.
A. (2023). Association between genetic polymorphism, severity, and treatment response among covid-19 infected egyptian
patients..  Frontiers in Pharmacology, , 14, 1209286. <https://dx.doi.org/10.3389/fphar.2023.1209286>
 
Link to the Ovid Full Text or citation: <https://ovidsp.ovid.com/ovidweb.cgi?T=JS&CSC=Y&NEWS=N&PAGE=fulltext&D=pmnm&AN=37426824>
 

Alarcao, V., Candeias, P., Stefanovska-Petkovska, M., Pintassilgo, S., Machado, F. L., Virgolino, A., Santos, O. (2023).
Mental health and well-being of migrant populations in portugal two years after the covid-19 pandemic..  Behavioral
sciences, , 13(5), . <https://dx.doi.org/10.3390/bs13050422>
 
Link to the Ovid Full Text or citation: <https://ovidsp.ovid.com/ovidweb.cgi?T=JS&CSC=Y&NEWS=N&PAGE=fulltext&D=pmnm&AN=37232659>
 

Alqahtani, T. M. T., Alelyani, A. A., Yousuf, M. M. M., Alhujayri, W. M. K., Husain, F. M., Zubair, M. (2023). Study of
plasmid-mediated extended-spectrum beta-lactamase-producing clinical strains of enterobacteriaceae from tabuk region..
Cureus, , 15(6), e40183. <https://dx.doi.org/10.7759/cureus.40183>
 
Link to the Ovid Full Text or citation: <https://ovidsp.ovid.com/ovidweb.cgi?T=JS&CSC=Y&NEWS=N&PAGE=fulltext&D=pmnm&AN=37431354>
 

An, Z., Nie, J., Huang, Y., Fang, P., Meng, X., Cai, C., Yu, L. (2023). Contribution of non-socioeconomic factors to
healthy quality of life in socioeconomically deprived patients with advanced gastrointestinal cancer: measuring
attributable fraction..  Journal of Advanced Nursing, , . <https://dx.doi.org/10.1111/jan.15862>
 
Link to the Ovid Full Text or citation: <https://ovidsp.ovid.com/ovidweb.cgi?T=JS&CSC=Y&NEWS=N&PAGE=fulltext&D=medp&AN=37710401>
 

Ariyawansa, S., Gunawardana, K. N., Hapudeniya, M. M., Manelgamage, N. J., Karunarathne, C. R., Madalagama, R. P.,
Ubeyratne, K. H., Wickramasinghe, D., Tun, H. M., Wu, P., Lam, T. T. Y., Chan, O. S. K. (2023). One health surveillance
of antimicrobial use and resistance: challenges and successes of implementing surveillance programs in sri lanka..
Antibiotics, , 12(3), . <https://dx.doi.org/10.3390/antibiotics12030446>
 
Link to the Ovid Full Text or citation: <https://ovidsp.ovid.com/ovidweb.cgi?T=JS&CSC=Y&NEWS=N&PAGE=fulltext&D=pmnm&AN=36978313>
 

Asperges, E., Albi, G., Zuccaro, V., Sambo, M., Pieri, T. C., Calia, M., Colaneri, M., Maiocchi, L., Melazzini, F.,
Lasagna, A., Peri, A., Mojoli, F., Sacchi, P., Bruno, R. (2023). Dynamic nlr and plr in predicting covid-19 severity: a
retrospective cohort study..  Infectious Diseases & Therapy, , 12(6), 1625-1640.
<https://dx.doi.org/10.1007/s40121-023-00813-1>
 
Link to the Ovid Full Text or citation: <https://ovidsp.ovid.com/ovidweb.cgi?T=JS&CSC=Y&NEWS=N&PAGE=fulltext&D=pmnm&AN=37198387>
 

Barber, C., Syski, A., Leaird, J., Call, R. C., Williams, A., Learn, P. (2021). Evaluating the efficacy of a screening
protocol for severe acute respiratory syndrome coronavirus 2 virus in asymptomatic preoperative/preprocedural patients
at a military hospital..  Military Medicine, , . <https://dx.doi.org/10.1093/milmed/usab522>
 
Link to the Ovid Full Text or citation: <https://ovidsp.ovid.com/ovidweb.cgi?T=JS&CSC=Y&NEWS=N&PAGE=fulltext&D=medp&AN=34894142>
 
Barrett, R. (2022). A cross-sectional study on substandard and falsified medicines (fake or counterfeit drugs) in uk
pharmacies during the covid-19 pandemic..  Expert Opinion on Drug Safety, , 1-11.
<https://dx.doi.org/10.1080/14740338.2023.2147922>
 
Link to the Ovid Full Text or citation: <https://ovidsp.ovid.com/ovidweb.cgi?T=JS&CSC=Y&NEWS=N&PAGE=fulltext&D=medp&AN=36374122>
 

Bendella, Z., Widmann, C. N., Layer, J. P., Layer, Y. L., Haase, R., Sauer, M., Bieler, L., Lehnen, N. C., Paech, D.,
Heneka, M. T., Radbruch, A., Schmeel, F. C. (2023). Brain volume changes after covid-19 compared to healthy controls by
artificial intelligence-based mri volumetry..  Diagnostics, , 13(10), . <https://dx.doi.org/10.3390/diagnostics13101716>
 
Link to the Ovid Full Text or citation: <https://ovidsp.ovid.com/ovidweb.cgi?T=JS&CSC=Y&NEWS=N&PAGE=fulltext&D=pmnm&AN=37238200>
 


Bhowmick, S., Mistri, T. K., Khan, M. R., Patil, P. C., Busquets, R., Ashif Ikbal, A. M., Choudhury, A., Roy, D. K.,
Palit, P., Saha, A. (2023). Investigation of bio-active amaryllidaceae alkaloidal small molecules as putative sars-cov-2
main protease and host tmprss2 inhibitors: interpretation by in-silico simulation study..  Journal of Biomolecular
Structure & Dynamics, , 1-21. <https://dx.doi.org/10.1080/07391102.2023.2238065>
 
Link to the Ovid Full Text or citation: <https://ovidsp.ovid.com/ovidweb.cgi?T=JS&CSC=Y&NEWS=N&PAGE=fulltext&D=medp&AN=37482789>
 


Blair, P. W., Siddharthan, T., Liu, G., Bai, J., Cui, E., East, J., Herrera, P., Anova, L., Mahadevan, V., Hwang, J.,
Hossen, S., Seo, S., Sonuga, O., Lawrence, J., Peters, J., Cox, A. L., Manabe, Y. C., Fenstermacher, K., Shea, S.,
Rothman, R. E., Hansoti, B., Sauer, L., Crainiceanu, C., Clark, D. V. (2022). Point-of-care lung ultrasound predicts
severe disease and death due to covid-19: a prospective cohort study..  Critical Care Explorations, , 4(8), e0732.
<https://dx.doi.org/10.1097/CCE.0000000000000732>
 
Link to the Ovid Full Text or citation: <https://ovidsp.ovid.com/ovidweb.cgi?T=JS&CSC=Y&NEWS=N&PAGE=fulltext&D=pmnm&AN=35982837>
 


Burns, J., Rivers, P., LeClair, L. B., Jovel, K. S., Rai, R. P., Lowe, A. A., Edwards, L. J., Khan, S. M., Mathenge, C.,
Ferraris, M., Kuntz, J. L., Lamberte, J. M., Hegmann, K. T., Odean, M. J., McLeland-Wieser, H., Beitel, S., Odame-Bamfo,
L., Schaefer Solle, N., Mak, J., Phillips, A. L., Sokol, B. E., Hollister, J., Ochoa, J. S., Grant, L., Thiese, M. S.,
Jacoby, K. B., Lutrick, K., Pubillones, F. A., Yoo, Y. M., Rentz Hunt, D., Ellingson, K., Berry, M. C., Gerald, J. K.,
Lopez, J., Gerald, L. B., Wesley, M. G., Krupp, K., Herring, M. K., Madhivanan, P., Caban-Martinez, A. J., Tyner, H. L.,
Meece, J. K., Yoon, S. K., Fowlkes, A. L., Naleway, A. L., Gwynn, L., Burgess, J. L., Thompson, M. G., Olsho, L. E.,
Gaglani, M. (2022). Pediatric research observing trends and exposures in covid-19 timelines (protect): protocol for a
multisite longitudinal cohort study..  JMIR Research Protocols, , 11(7), e37929. <https://dx.doi.org/10.2196/37929>
 
Link to the Ovid Full Text or citation: <https://ovidsp.ovid.com/ovidweb.cgi?T=JS&CSC=Y&NEWS=N&PAGE=fulltext&D=pmnm&AN=35635842>
 


Byambasuren, O., Stehlik, P., Clark, J., Alcorn, K., Glasziou, P. (2023). Effect of covid-19 vaccination on long covid:
systematic review..  BMJ Medicine, , 2(1), e000385. <https://dx.doi.org/10.1136/bmjmed-2022-000385>
 
Link to the Ovid Full Text or citation: <https://ovidsp.ovid.com/ovidweb.cgi?T=JS&CSC=Y&NEWS=N&PAGE=fulltext&D=pmnm&AN=36936268>
 


Camargo Junior, E. B., Noivo, I. S., Gouvea, T. C. C., Fernandes, M. N. F., Gherardi-Donato, E. C. D. S. (2023).
Depression and substance use among brazilian university students during the covid-19 pandemic..  Journal of Psychoactive
Drugs, , 1-10. <https://dx.doi.org/10.1080/02791072.2023.2244499>
 
Link to the Ovid Full Text or citation: <https://ovidsp.ovid.com/ovidweb.cgi?T=JS&CSC=Y&NEWS=N&PAGE=fulltext&D=medp&AN=37551709>
 


Cegolon, L., Negro, C., Pesce, M., Filon, F. L. (2023). Covid-19 incidence and vaccine effectiveness in university
staff, 1 march 2020-2 april 2022..  101629355, , 11(2), . <https://dx.doi.org/10.3390/vaccines11020483>
 
Link to the Ovid Full Text or citation: <https://ovidsp.ovid.com/ovidweb.cgi?T=JS&CSC=Y&NEWS=N&PAGE=fulltext&D=pmnm&AN=36851360>
 


Cena, T., Cammarota, G., Azzolina, D., Barini, M., Bazzano, S., Zagaria, D., Negroni, D., Castello, L., Carriero, A.,
Corte, F. D., Vaschetto, R. (2021). Predictors of intubation and mortality in covid-19 patients: a retrospective study..
 Journal of Anesthesia, Analgesia and Critical Care (Online), , 1(1), 19. <https://dx.doi.org/10.1186/s44158-021-00016-5>
 
Link to the Ovid Full Text or citation: <https://ovidsp.ovid.com/ovidweb.cgi?T=JS&CSC=Y&NEWS=N&PAGE=fulltext&D=pmnm&AN=37386623>
 


Clayton, N. A., Ward, E. C., Norman, E., Ryan, H., Kol, M. R. (2023). Speech pathology assessment of dysphagia post
endotracheal extubation: a service-model evaluation..  Australian Critical Care, , .
<https://dx.doi.org/10.1016/j.aucc.2023.07.041>
 
Link to the Ovid Full Text or citation: <https://ovidsp.ovid.com/ovidweb.cgi?T=JS&CSC=Y&NEWS=N&PAGE=fulltext&D=medp&AN=37709658>
 


Clofent, D., Culebras, M., Felipe-Montiel, A., Arjona-Peris, M., Granados, G., Saez, M., Pilia, F., Ferreiro, A.,
Alvarez, A., Loor, K., Bosch-Nicolau, P., Polverino, E. (2023). Serial lung ultrasound in monitoring viral pneumonia:
the lesson learned from covid-19..  Erj Open Research, , 9(4), . <https://dx.doi.org/10.1183/23120541.00017-2023>
 
Link to the Ovid Full Text or citation: <https://ovidsp.ovid.com/ovidweb.cgi?T=JS&CSC=Y&NEWS=N&PAGE=fulltext&D=pmnm&AN=37583967>
 


Czuba, B., Mlodawski, J., Kajdy, A., Sys, D., Cnota, W., Mlodawska, M., Kwiatkowski, S., Guzik, P., Wielgos, M.,
Rybak-Krzyszkowska, M., Fuchs, A., Swiercz, G., Borowski, D. (2022). Implementation of the publicly funded prenatal
screening programme in poland during the covid-19 pandemic: a cross-sectional study..  Journal of Clinical Medicine, ,
11(5), . <https://dx.doi.org/10.3390/jcm11051317>
 
Link to the Ovid Full Text or citation: <https://ovidsp.ovid.com/ovidweb.cgi?T=JS&CSC=Y&NEWS=N&PAGE=fulltext&D=pmnm&AN=35268408>
 


De Nardo, P., Tebon, M., Savoldi, A., Soriolo, N., Danese, E., Peserico, D., Morra, M., Gentilotti, E., Caliskan, G.,
Marchetti, P., Cecchetto, R., Mazzariol, A., Verlato, G., Gibellini, D., Tacconelli, E. (2023). Diagnostic accuracy of a
rapid sars-cov-2 antigen test among people experiencing homelessness: a prospective cohort and implementation study..
Infectious Diseases & Therapy, , 12(4), 1073-1082. <https://dx.doi.org/10.1007/s40121-023-00787-0>
 
Link to the Ovid Full Text or citation: <https://ovidsp.ovid.com/ovidweb.cgi?T=JS&CSC=Y&NEWS=N&PAGE=fulltext&D=pmnm&AN=36907951>
 


Derman, W., Runciman, P., Eken, M., Boer, P. H., Blauwet, C., Bogdos, M., Idrisova, G., Jordaan, E., Kissick, J., LeVan,
P., Lexell, J., Mohammadi, F., Patricio, M., Schwellnus, M., Webborn, N., Willick, S. E., Yagishita, K. (2022).
Incidence and burden of illness at the tokyo 2020 paralympic games held during the covid-19 pandemic: a prospective
cohort study of 66 045 athlete days..  British Journal of Sports Medicine, , .
<https://dx.doi.org/10.1136/bjsports-2022-106312>
 
Link to the Ovid Full Text or citation: <https://ovidsp.ovid.com/ovidweb.cgi?T=JS&CSC=Y&NEWS=N&PAGE=fulltext&D=medp&AN=36588431>
 


Dhanasekaran, K., Hariprasad, R., Singh, M., Jain, S., Nethan, S. T., Singh, S. (2023). Impact of the covid-19 pandemic
on an online cancer screening training programme for healthcare providers in the public sector in india: learnings from
a hub and spoke model perspective..  Ecancermedicalscience, , 17, 1513. <https://dx.doi.org/10.3332/ecancer.2023.1513>
 
Link to the Ovid Full Text or citation: <https://ovidsp.ovid.com/ovidweb.cgi?T=JS&CSC=Y&NEWS=N&PAGE=fulltext&D=pmnm&AN=37113710>
 

Divo, M. J., Liu, C., Polverino, F., Castaldi, P. J., Celli, B. R., Tesfaigzi, Y. (2023). From pre-copd to copd: a
simple, low cost, and ease of implementation (slim) risk calculator..  European Respiratory Journal, , .
<https://dx.doi.org/10.1183/13993003.00806-2023>
 
Link to the Ovid Full Text or citation: <https://ovidsp.ovid.com/ovidweb.cgi?T=JS&CSC=Y&NEWS=N&PAGE=fulltext&D=medp&AN=37678951>
 

Dubar, V., Pascreau, T., Dupont, A., Dubucquoi, S., Dautigny, A. L., Ghozlan, B., Zuber, B., Mellot, F., Vasse, M.,
Susen, S., Poissy, J., Gaudet, A. (2023). Development of a decision support tool for anticoagulation in critically ill
patients admitted for sars-cov-2 infection: the calt protocol..  Biomedicines, , 11(6), .
<https://dx.doi.org/10.3390/biomedicines11061504>
 
Link to the Ovid Full Text or citation: <https://ovidsp.ovid.com/ovidweb.cgi?T=JS&CSC=Y&NEWS=N&PAGE=fulltext&D=pmnm&AN=37371599>


Dutta, P., Islam, A., Sayeed, M. A., Rahman, M. A., Abdullah, M. S., Saha, O., Rahman, M. Z., Klaassen, M., Hoque, M.
A., Hassan, M. M. (2022). Epidemiology and molecular characterization of avian influenza virus in backyard poultry of
chattogram, bangladesh..  Infection, Genetics & Evolution, , 105, 105377. <https://dx.doi.org/10.1016/j.meegid.2022.105377>
 
Link to the Ovid Full Text or citation: <https://ovidsp.ovid.com/ovidweb.cgi?T=JS&CSC=Y&NEWS=N&PAGE=fulltext&D=medp&AN=36220485>
 

Edwards, L. J., Fowlkes, A. L., Wesley, M. G., Kuntz, J. L., Odean, M. J., Caban-Martinez, A. J., Dunnigan, K.,
Phillips, A. L., Grant, L., Herring, M. K., Groom, H. C., Respet, K., Beitel, S., Zunie, T., Hegmann, K. T., Kumar, A.,
Joseph, G., Poe, B., Louzado-Feliciano, P., Smith, M. E., Thiese, M. S., Schaefer-Solle, N., Yoo, Y. M., Silvera, C. A.,
Mayo Lamberte, J., Mak, J., McDonald, L. C., Stuckey, M. J., Kutty, P., Arvay, M. L., Yoon, S. K., Tyner, H. L.,
Burgess, J. L., Hunt, D. R., Meece, J., Gaglani, M., Naleway, A. L., Thompson, M. G. (2021). Research on the
epidemiology of sars-cov-2 in essential response personnel (recover): protocol for a multisite longitudinal cohort
study..  JMIR Research Protocols, , 10(12), e31574. <https://dx.doi.org/10.2196/31574>
 
Link to the Ovid Full Text or citation: <https://ovidsp.ovid.com/ovidweb.cgi?T=JS&CSC=Y&NEWS=N&PAGE=fulltext&D=pmnm&AN=34662287>
 

El-Ghitany, E. M., Ashour, A., Elmorshedy, H., Farghaly, A. G., Hashish, M. H., Omran, E. A. (2023). Adherence of
healthcare workers to covid-19 infection prevention practices and its relationship with sars-cov-2 seropositivity..
Journal of Infection Prevention, , 24(3), 119-131. <https://dx.doi.org/10.1177/17571774231158782>
 
Link to the Ovid Full Text or citation: <https://ovidsp.ovid.com/ovidweb.cgi?T=JS&CSC=Y&NEWS=N&PAGE=fulltext&D=pmnm&AN=37051305>
 
El-Khatib, Z., Richter, L., Reich, A., Benka, B., Assadian, O. (2023). Implementation of a surveillance system for
severe acute respiratory infections at a tertiary care hospital in austria: protocol for a retrospective longitudinal
feasibility study..  JMIR Research Protocols, , 12, e47547. <https://dx.doi.org/10.2196/47547>
 
Link to the Ovid Full Text or citation: <https://ovidsp.ovid.com/ovidweb.cgi?T=JS&CSC=Y&NEWS=N&PAGE=fulltext&D=pmnm&AN=37535414>
 
Eligulashvili, A., Darrell, M., Miller, C., Lee, J., Congdon, S., Lee, J. S., Hsu, K., Yee, J., Hou, W., Islam, M.,
Duong, T. Q. (2022). Covid-19 patients in the covid-19 recovery and engagement (core) clinics in the bronx..
Diagnostics, , 13(1), . <https://dx.doi.org/10.3390/diagnostics13010119>
 
Link to the Ovid Full Text or citation: <https://ovidsp.ovid.com/ovidweb.cgi?T=JS&CSC=Y&NEWS=N&PAGE=fulltext&D=pmnm&AN=36611411>
 
Farrow, L., Ashcroft, G. P., Zhong, M., Anderson, L. (2022). Using artificial intelligence to revolutionise the patient
care pathway in hip and knee arthroplasty (archery): protocol for the development of a clinical prediction model..  JMIR
Research Protocols, , 11(5), e37092. <https://dx.doi.org/10.2196/37092>
 
Link to the Ovid Full Text or citation: <https://ovidsp.ovid.com/ovidweb.cgi?T=JS&CSC=Y&NEWS=N&PAGE=fulltext&D=pmnm&AN=35544289>
 
Faux-Nightingale, A., Burton, C., Twohig, H., Blagojevic-Bucknall, M., Carroll, W., Chew-Graham, C. A., Dunn, K.,
Gilchrist, F., Helliwell, T., Lawton, O., Lawton, S., Mallen, C., Saunders, B., van der Windt, D., Welsh, V. (2023).
Symptom patterns and life with post-acute covid-19 in children aged 8-17 years: a mixed-methods study protocol..  Bjgp
Open, , 7(2), . <https://dx.doi.org/10.3399/BJGPO.2022.0149>
 
Link to the Ovid Full Text or citation: <https://ovidsp.ovid.com/ovidweb.cgi?T=JS&CSC=Y&NEWS=N&PAGE=fulltext&D=pmnm&AN=36759021>
 
Feijt, M., de Kort, Y., Westerink, J., Bierbooms, J., Bongers, I., IJsselsteijn, W. (2022). Integrating technology in
mental healthcare practice: a repeated cross-sectional survey study on professionals' adoption of digital mental health
before and during covid-19..  Frontiers in psychiatry Frontiers Research Foundation, , 13, 1040023.
<https://dx.doi.org/10.3389/fpsyt.2022.1040023>
 
Link to the Ovid Full Text or citation: <https://ovidsp.ovid.com/ovidweb.cgi?T=JS&CSC=Y&NEWS=N&PAGE=fulltext&D=pmnm&AN=36874171>
 
Gerges, S., Obeid, S., Hallit, S. (2023). Traversing mental health disorders during pregnancy: lebanese women's
experiences of antepartum depression and anxiety..  Irish Journal of Medical Science, , .
<https://dx.doi.org/10.1007/s11845-023-03371-0>
 
Link to the Ovid Full Text or citation: <https://ovidsp.ovid.com/ovidweb.cgi?T=JS&CSC=Y&NEWS=N&PAGE=fulltext&D=medp&AN=37081286>


Guchhait, P., Chaudhuri, B. N., Das, S. (2023). Bloodstream infections with opportunistic pathogens in covid-19 era: a
real challenge necessitates stringent infection control..  Journal of Laboratory Physicians, , 15(1), 131-138.
<https://dx.doi.org/10.1055/s-0043-1764476>
 
Link to the Ovid Full Text or citation: <https://ovidsp.ovid.com/ovidweb.cgi?T=JS&CSC=Y&NEWS=N&PAGE=fulltext&D=pmnm&AN=37064967>
 
Hammett, D. L., Loiselle, C., Palmer, K. M., Loiselle, J. M., Attia, M. W. (2023). Covid-19 screening in the pediatric
emergency department..  Cureus, , 15(3), e35731. <https://dx.doi.org/10.7759/cureus.35731>
 
Link to the Ovid Full Text or citation: <https://ovidsp.ovid.com/ovidweb.cgi?T=JS&CSC=Y&NEWS=N&PAGE=fulltext&D=pmnm&AN=37016637>
 
Han, J. G., Sun, L. T., Zhai, Z. W., Xia, P. D., Hu, H., Zhang, D., Jiang, C. Q., Zhao, B. C., Qu, H., Qian, Q., Dai,
Y., Yao, H. W., Wang, Z. J. (2023). [the value of transanal multipoint full-layer puncture biopsy in determining the
response degree of rectal cancer following neoadjuvant therapy: a prospective multicenter study]..  Chung-Hua Wai Ko Tsa
Chih [Chinese Journal of Surgery], , 61(9), 769-776. <https://dx.doi.org/10.3760/cma.j.cn112139-20230417-00170>
 
Link to the Ovid Full Text or citation: <https://ovidsp.ovid.com/ovidweb.cgi?T=JS&CSC=Y&NEWS=N&PAGE=fulltext&D=medp&AN=37491169>
 
Hanratty, J., Keenan, C., O'Connor, S. R., Leonard, R., Chi, Y., Ferguson, J., Axiaq, A., Miller, S., Bradley, D.,
Dempster, M. (2023). Psychological and psychosocial determinants of covid health related behaviours (cohere): an
evidence and gap map..  Campbell Systematic Reviews, , 19(3), e1336. <https://dx.doi.org/10.1002/cl2.1336>
 
Link to the Ovid Full Text or citation: <https://ovidsp.ovid.com/ovidweb.cgi?T=JS&CSC=Y&NEWS=N&PAGE=fulltext&D=pmnm&AN=37361553>

Hohl, S. D., Maxwell, A. E., Sharma, K. P., Sun, J., Vu, T. T., DeGroff, A., Escoffery, C., Schlueter, D., Hannon, P. A.
(2023). Implementing mailed colorectal cancer fecal screening tests in real-world primary care settings: promising
implementation practices and opportunities for improvement..  Prevention Science, , .
<https://dx.doi.org/10.1007/s11121-023-01496-3>
 
Link to the Ovid Full Text or citation: <https://ovidsp.ovid.com/ovidweb.cgi?T=JS&CSC=Y&NEWS=N&PAGE=fulltext&D=medp&AN=36952143>
 
Howard, M., Aubrey-Bassler, K., Drummond, N., Lussier, M. T., Queenan, J. A., Vanstone, M., Nicholson, K., Ramdyal, A.,
Lawson, J., Hafid, S., Freeman, K., Clark, R., Mangin, D. (2023). Effects of the covid-19 pandemic on primary health
care for chronic conditions in canada: protocol for a retrospective pre-post study using national practice-based
research network data..  JMIR Research Protocols, , 12, e49131. <https://dx.doi.org/10.2196/49131>
 
Link to the Ovid Full Text or citation: <https://ovidsp.ovid.com/ovidweb.cgi?T=JS&CSC=Y&NEWS=N&PAGE=fulltext&D=pmnm&AN=37477967>
 
Hui, S., Sane, N., Wang, A., Wan, L., Bell, S., Le, S., Dev, A. (2023). Hepatocellular carcinoma surveillance in the
telehealth era: a single-centre review..  Journal of Telemedicine & Telecare, , 1357633X231166032.
<https://dx.doi.org/10.1177/1357633X231166032>
 
Link to the Ovid Full Text or citation: <https://ovidsp.ovid.com/ovidweb.cgi?T=JS&CSC=Y&NEWS=N&PAGE=fulltext&D=medp&AN=37032467>
 
Javorszky, S. M., Reiter, R., Iglseder, B. (2023). Validation of a geriatric bedside swallowing screen (gebs): protocol
of a prospective cohort study..  JMIR Research Protocols, , 12, e46252. <https://dx.doi.org/10.2196/46252>
Link to the Ovid Full Text or citation: <https://ovidsp.ovid.com/ovidweb.cgi?T=JS&CSC=Y&NEWS=N&PAGE=fulltext&D=pmnm&AN=37566452>


Jovanovic, C. E. S., Kalam, F., Granata, F., Pfammatter, A. F., Spring, B. (2022). Validation and results of a novel
survey assessing decisional balance for a whole food plant-based diet among us adults..  Frontiers in Nutrition, , 9,
958611. <https://dx.doi.org/10.3389/fnut.2022.958611>
 
Link to the Ovid Full Text or citation: <https://ovidsp.ovid.com/ovidweb.cgi?T=JS&CSC=Y&NEWS=N&PAGE=fulltext&D=pmnm&AN=36245546>
 
Kawuki, J., Fang, Y., Yu, F. Y., Ye, D., Chan, P. S., Chen, S., Wang, Z. (2023). Facilitators and barriers to use rapid
antigen test for sars-cov-2 among community-dwelling older adults in hong kong: a population-based cross-sectional
random telephone survey..  PLOS Global Public Health, , 3(8), e0002196. <https://dx.doi.org/10.1371/journal.pgph.0002196>
 
Link to the Ovid Full Text or citation: <https://ovidsp.ovid.com/ovidweb.cgi?T=JS&CSC=Y&NEWS=N&PAGE=fulltext&D=pmnm&AN=37552663>
 
Khamadi, S. A., Mavere, C., Bahemana, E., Lwilla, A., Mizinduko, M., Bwigane, S., Peter, A., Makando, J., Peter, B.,
Agaba, P., Shah, N., Julu, B., Ganesan, K., Coakley, P., Lee, E. H. (2023). Early warning indicators of hiv drug
resistance in the southern highlands region of tanzania: lessons from a cross-sectional surveillance study..  PLOS
Global Public Health, , 3(3), e0000929. <https://dx.doi.org/10.1371/journal.pgph.0000929>
 
Link to the Ovid Full Text or citation: <https://ovidsp.ovid.com/ovidweb.cgi?T=JS&CSC=Y&NEWS=N&PAGE=fulltext&D=pmnm&AN=36996096>

Kostka, K., Duarte-Salles, T., Prats-Uribe, A., Sena, A. G., Pistillo, A., Khalid, S., Lai, L. Y. H., Golozar, A.,
Alshammari, T. M., Dawoud, D. M., Nyberg, F., Wilcox, A. B., Andryc, A., Williams, A., Ostropolets, A., Areia, C., Jung,
C. Y., Harle, C. A., Reich, C. G., Blacketer, C., Morales, D. R., Dorr, D. A., Burn, E., Roel, E., Tan, E. H., Minty,
E., DeFalco, F., de Maeztu, G., Lipori, G., Alghoul, H., Zhu, H., Thomas, J. A., Bian, J., Park, J., Martinez Roldan,
J., Posada, J. D., Banda, J. M., Horcajada, J. P., Kohler, J., Shah, K., Natarajan, K., Lynch, K. E., Liu, L.,
Schilling, L. M., Recalde, M., Spotnitz, M., Gong, M., Matheny, M. E., Valveny, N., Weiskopf, N. G., Shah, N., Alser,
O., Casajust, P., Park, R. W., Schuff, R., Seager, S., DuVall, S. L., You, S. C., Song, S., Fernandez-Bertolin, S.,
Fortin, S., Magoc, T., Falconer, T., Subbian, V., Huser, V., Ahmed, W. U., Carter, W., Guan, Y., Galvan, Y., He, X.,
Rijnbeek, P. R., Hripcsak, G., Ryan, P. B., Suchard, M. A., Prieto-Alhambra, D. (2022). Unraveling covid-19: a
large-scale characterization of 4.5 million covid-19 cases using charybdis..  Clinical Epidemiology, , 14, 369-384.
<https://dx.doi.org/10.2147/CLEP.S323292>
 
Link to the Ovid Full Text or citation: <https://ovidsp.ovid.com/ovidweb.cgi?T=JS&CSC=Y&NEWS=N&PAGE=fulltext&D=pmnm&AN=35345821>
 
Lafay, C., Assad, Z., Ouldali, N., Quoc, E. B., Clement, A., Durand, C., Fares, S., Faye, A., Eveillard, L. A.,
Kaguelidou, F., Titah, C., Valtuille, Z., Vinit, C., Meinzer, U., Dumaine, C. (2023). Increased incidence of pediatric
uveitis associated with the covid-19 pandemic occurring before covid-19 vaccine implementation: a time-series analysis..
 Journal of Pediatrics, , 263, 113682. <https://dx.doi.org/10.1016/j.jpeds.2023.113682>
 
Link to the Ovid Full Text or citation: <https://ovidsp.ovid.com/ovidweb.cgi?T=JS&CSC=Y&NEWS=N&PAGE=fulltext&D=medp&AN=37611738>
 
Laranjeira, C., Dixe, M. A., Querido, A. (2023). Mental health status and coping among portuguese higher education
students in the early phase of the covid-19 pandemic..  European Journal of Investigation in Health Psychology &
Education, , 13(2), 429-439. <https://dx.doi.org/10.3390/ejihpe13020032>
 
Link to the Ovid Full Text or citation: <https://ovidsp.ovid.com/ovidweb.cgi?T=JS&CSC=Y&NEWS=N&PAGE=fulltext&D=pmnm&AN=36826216>
 

Letourneau, N., McDonald, S., MacKay, L. J., Bell, R. C., Hetherington, E., Deane, A. J., Dewey, D., Edwards, S., Field,
C. J., Giesbrecht, G. F., Graham, S., Lebel, C., Leung, B., Madigan, S., McArthur, B. A., McMorris, C., Racine, N.,
Ross, K. M., Wu, M., Tough, S. C. (2021). Cross-sectional study protocol for the covid-19 impact survey of mothers and
their 7-11 year old children in alberta, canada..  Frontiers in psychiatry Frontiers Research Foundation, , 12, 597759.
<https://dx.doi.org/10.3389/fpsyt.2021.597759>
 
Link to the Ovid Full Text or citation: <https://ovidsp.ovid.com/ovidweb.cgi?T=JS&CSC=Y&NEWS=N&PAGE=fulltext&D=pmnm&AN=34239455>
 

Levante, A., Martis, C., Bianco, F., Castelli, I., Petrocchi, S., Lecciso, F. (2023). Internalizing and externalizing
symptoms in children during the covid-19 pandemic: a systematic mixed studies review..  Frontiers in Psychology, , 14,
1182309. <https://dx.doi.org/10.3389/fpsyg.2023.1182309>
 
Link to the Ovid Full Text or citation: <https://ovidsp.ovid.com/ovidweb.cgi?T=JS&CSC=Y&NEWS=N&PAGE=fulltext&D=pmnm&AN=37397311>


Lima, Y., Rice, S. (2022). Mental health symptoms and correlates among amateur football players: a cross-sectional
study..  International Journal of Environmental Health Research, , 1-12. <https://dx.doi.org/10.1080/09603123.2022.2112659>
 
Link to the Ovid Full Text or citation: <https://ovidsp.ovid.com/ovidweb.cgi?T=JS&CSC=Y&NEWS=N&PAGE=fulltext&D=medp&AN=35968854>
 
Lucijanic, M., Tjesic-Drinkovic, I., Piskac Zivkovic, N., Pastrovic, F., Rob, Z., Bacevac, M., Sedinic Lacko, M.,
Dzambas, E., Medic, B., Vukoja, I., Busic, I., Grgurevic, I., Luksic, I., Barsic, B. (2023). Incidence, risk factors and
mortality associated with major bleeding events in hospitalized covid-19 patients..  Life, , 13(8), .
<https://dx.doi.org/10.3390/life13081699>
 
Link to the Ovid Full Text or citation: <https://ovidsp.ovid.com/ovidweb.cgi?T=JS&CSC=Y&NEWS=N&PAGE=fulltext&D=pmnm&AN=37629556>
 
Lugo-Radillo, A., Mendoza-Cano, O., Trujillo, X., Huerta, M., Rios-Silva, M., Guzman-Esquivel, J., Benites-Godinez, V.,
Bricio-Barrios, J. A., Rios-Bracamontes, E. F., Cardenas-Rojas, M. I., Cardenas, Y., Murillo-Zamora, E. (2023).
Assessing the burden of dengue during the covid-19 pandemic in mexico..  Tropical Medicine & Infectious Disease, , 8(4),
. <https://dx.doi.org/10.3390/tropicalmed8040232>
 
Link to the Ovid Full Text or citation: <https://ovidsp.ovid.com/ovidweb.cgi?T=JS&CSC=Y&NEWS=N&PAGE=fulltext&D=pmnm&AN=37104357>
 


Lutrick, K., Ellingson, K. D., Baccam, Z., Rivers, P., Beitel, S., Parker, J., Hollister, J., Sun, X., Gerald, J. K.,
Komatsu, K., Kim, E., LaFleur, B., Grant, L., Yoo, Y. M., Kumar, A., Mayo Lamberte, J., Cowling, B. J., Cobey, S.,
Thornburg, N. J., Meece, J. K., Kutty, P., Nikolich-Zugich, J., Thompson, M. G., Burgess, J. L. (2021). Covid-19
infection, reinfection, and vaccine effectiveness in a prospective cohort of arizona frontline/essential workers: the az
heroes research protocol..  JMIR Research Protocols, , . <https://dx.doi.org/10.2196/28925>
 
Link to the Ovid Full Text or citation: <https://ovidsp.ovid.com/ovidweb.cgi?T=JS&CSC=Y&NEWS=N&PAGE=fulltext&D=medp&AN=34057904>
 
Marzo, R. R., Khanal, P., Ahmad, A., Rathore, F. A., Chauhan, S., Singh, A., Shrestha, S., AlRifai, A., Lotfizadeh, M.,
Younus, D. A., Billah, M. A., Rahman, F., Sivaladchanam, Y., Mohan, D., Su, T. T. (2022). Quality of life of the elderly
during the covid-19 pandemic in asian countries: a cross-sectional study across six countries..  Life, , 12(3), .
<https://dx.doi.org/10.3390/life12030365>
 
Link to the Ovid Full Text or citation: <https://ovidsp.ovid.com/ovidweb.cgi?T=JS&CSC=Y&NEWS=N&PAGE=fulltext&D=pmnm&AN=35330116>
 
Metz, T. D., Clifton, R. G., Gallagher, R., Gross, R. S., Horwitz, L. I., Jacoby, V. L., Martin-Herz, S. P.,
Peralta-Carcelen, M., Reeder, H. T., Beamon, C. J., Bind, M. A., Chan, J., Chang, A. A., Chibnik, L. B., Costantine, M.
M., Fitzgerald, M. L., Foulkes, A. S., Gibson, K. S., Guthe, N., Habli, M., Hackney, D. N., Hoffman, M. K., Hoffman, M.
C., Hughes, B. L., Katz, S. D., Laleau, V., Mallett, G., Mendez-Figueroa, H., Monzon, V., Palatnik, A., Palomares, K. T.
S., Parry, S., Peralta-Carcelen, M., Pettker, C. M., Plunkett, B. A., Poppas, A., Reddy, U. M., Rouse, D. J., Saade, G.
R., Sandoval, G. J., Schlater, S. M., Sciurba, F. C., Simhan, H. N., Skupski, D. W., Sowles, A., Thaweethai, T., Thomas,
G. L., Thorp, J. M., Tita, A. T., Weiner, S. J., Weigand, S., Yee, L. M., Flaherman, V. J. (2023). Researching covid to
enhance recovery (recover) pregnancy study: rationale, objectives and design..  MedRxiv : the Preprint Server for Health
Sciences, , . <https://dx.doi.org/10.1101/2023.04.24.23289025>
 
Link to the Ovid Full Text or citation: <https://ovidsp.ovid.com/ovidweb.cgi?T=JS&CSC=Y&NEWS=N&PAGE=fulltext&D=pmnm&AN=37162923>
 
Mihevc, M., Petek Ster, M. (2023). Stigma and low sense of coherence as long-term predictors of depressive and anxiety
symptoms in the population amid the covid-19 pandemic..  Psychiatria Danubina, , 35(2), 250-259.
<https://dx.doi.org/10.24869/psyd.2023.250>
 
Link to the Ovid Full Text or citation: <https://ovidsp.ovid.com/ovidweb.cgi?T=JS&CSC=Y&NEWS=N&PAGE=fulltext&D=medp&AN=37480314>
 
Mizrahi Reuveni, M., Kertes, J., Shapiro Ben David, S., Shahar, A., Shamir-Stein, N., Rosen, K., Liran, O., Bar-Yishay,
M., Adler, L. (2023). Risk stratification model for severe covid-19 disease: a retrospective cohort study..
Biomedicines, , 11(3), . <https://dx.doi.org/10.3390/biomedicines11030767>
 
Link to the Ovid Full Text or citation: <https://ovidsp.ovid.com/ovidweb.cgi?T=JS&CSC=Y&NEWS=N&PAGE=fulltext&D=pmnm&AN=36979745>
 
Morish, A., Alsaigh, A., Almaghrabi, E., Alenzi, H., Ahmed, N. M., AlAhdal, A. (2023). Quality performance evaluation of
the largest covid-19-designated intensive care unit in the western region of saudi arabia..  Cureus, , 15(5), e39800.
<https://dx.doi.org/10.7759/cureus.39800>
 
Link to the Ovid Full Text or citation: <https://ovidsp.ovid.com/ovidweb.cgi?T=JS&CSC=Y&NEWS=N&PAGE=fulltext&D=pmnm&AN=37398805>
 
Moussa, F. L., Moussa, M. L., Alharbi, H. A., Omer, T., Sofiany, H. A., Oqdi, Y. A., Alblowi, B. H., Alblowi, S. H.
(2023). Telehealth readiness of healthcare providers during covid-19 pandemic in saudi arabia..  Healthcare, , 11(6), .
<https://dx.doi.org/10.3390/healthcare11060842>
 
Link to the Ovid Full Text or citation: <https://ovidsp.ovid.com/ovidweb.cgi?T=JS&CSC=Y&NEWS=N&PAGE=fulltext&D=pmnm&AN=36981499>
 
Nguyen, H. L., Thai, N. Q., Li, M. S. (2023). Identifying inhibitors of nsp16-nsp10 of sars-cov-2 from large databases..
 Journal of Biomolecular Structure & Dynamics, , 41(15), 7045-7054. <https://dx.doi.org/10.1080/07391102.2022.2114941>
 
Link to the Ovid Full Text or citation: <https://ovidsp.ovid.com/ovidweb.cgi?T=JS&CSC=Y&NEWS=N&PAGE=fulltext&D=pmnm&AN=36002258>
 

O'Byrne, L., Gavin, B., Adamis, D., Lim, Y. X., McNicholas, F. (2021). Levels of stress in medical students due to
covid-19..  Journal of Medical Ethics, , . <https://dx.doi.org/10.1136/medethics-2020-107155>
 
Link to the Ovid Full Text or citation: <https://ovidsp.ovid.com/ovidweb.cgi?T=JS&CSC=Y&NEWS=N&PAGE=fulltext&D=medp&AN=33658333>
 

Ogboghodo, E. O., Osaigbovo, I. I., Obaseki, D. E., Iduitua, M. T. N., Asamah, D., Oduware, E., Okwara, B. U. (2022).
Implementation of a covid-19 screening tool in a southern nigerian tertiary health facility..  PLOS Global Public
Health, , 2(8), e0000578. <https://dx.doi.org/10.1371/journal.pgph.0000578>
 
Link to the Ovid Full Text or citation: <https://ovidsp.ovid.com/ovidweb.cgi?T=JS&CSC=Y&NEWS=N&PAGE=fulltext&D=pmnm&AN=36962763>
 
Oliveira, L. B., Souza, L. M., Lima, F. M., Fhon, J. R. S., Puschel, V. A. A., Carbogim, F. D. C. (2022). Factors
associated with the illness of nursing professionals caused by covid-19 in three university hospitals in brazil..  Sh@w,
, 13(2), 255-260. <https://dx.doi.org/10.1016/j.shaw.2022.03.001>
 
Link to the Ovid Full Text or citation: <https://ovidsp.ovid.com/ovidweb.cgi?T=JS&CSC=Y&NEWS=N&PAGE=fulltext&D=pmnm&AN=35309963>
 
Pan, N., Liu, Y., Zhang, H., Xu, Y., Bao, X., Sheng, S., Liang, Y., Liu, B., Lyu, Y., Li, H., Ma, F., Pan, H., Wang, X.
(2023). Oral vaccination with engineered probiotic limosilactobacillus reuteri has protective effects against localized
and systemic staphylococcus aureus infection..  Microbiology Spectrum, , e0367322.
<https://dx.doi.org/10.1128/spectrum.03673-22>
 
Link to the Ovid Full Text or citation: <https://ovidsp.ovid.com/ovidweb.cgi?T=JS&CSC=Y&NEWS=N&PAGE=fulltext&D=medp&AN=36723073>
 


Patte, K. A., Battista, K., Ferro, M. A., Belanger, R. E., Wade, T. J., Faulkner, G., Pickett, W., Riazi, N. A.,
Michaelson, V., Carsley, S., Leatherdale, S. T. (2023). School learning modes during the covid-19 response and pre- to
during pandemic mental health changes in a prospective cohort of canadian adolescents..  Social Psychiatry & Psychiatric
Epidemiology, , . <https://dx.doi.org/10.1007/s00127-023-02557-2>
 
Link to the Ovid Full Text or citation: <https://ovidsp.ovid.com/ovidweb.cgi?T=JS&CSC=Y&NEWS=N&PAGE=fulltext&D=medp&AN=37668673>
 
Peiro Morant, J. F., Ramirez Torres, J. M., Perez Vazquez, E., Lozano Bouzon, V. M., Parra Valderrama, A., Frias Vargas,
M., en nombre del Grupo de Trabajo de Vasculopatias de SEMERGEN (2023). [knowledge of chronic venous disease among
healthcare professionals in spain]..  Semergen Sociedad Espanola de Medicina Rural y Generalista, , 49(8), 102063.
<https://dx.doi.org/10.1016/j.semerg.2023.102063>
 
Link to the Ovid Full Text or citation: <https://ovidsp.ovid.com/ovidweb.cgi?T=JS&CSC=Y&NEWS=N&PAGE=fulltext&D=medp&AN=37619267>
 
Perez-Jacoiste Asin, M. A., De Castro, M., De Dios, B., Perez-Ayala, A., Lalueza, A., Garcia-Reyne, A., Losada, I.,
Herrero-Martinez, J. M., Jimenez, P. H., Lumbreras, C., Lizasoain, M., Lopez Medrano, F. (2023). Enhancing screening
adherence for strongyloides infection in latinx inpatients with covid-19: a local protocol implementation study..
Pathogens and Global Health, , 1-9. <https://dx.doi.org/10.1080/20477724.2023.2240088>
 
Link to the Ovid Full Text or citation: <https://ovidsp.ovid.com/ovidweb.cgi?T=JS&CSC=Y&NEWS=N&PAGE=fulltext&D=medp&AN=37525476>

Peskar, M., Simunic, B., Slosar, L., Pisot, S., Teraz, K., Gasparini, M., Pisot, R., Marusic, U. (2023). Effects of
covid-19 on cognition and mood after hospitalization and at 2-month follow-up..  Frontiers in Psychology, , 14, 1141809.
<https://dx.doi.org/10.3389/fpsyg.2023.1141809>
 
Link to the Ovid Full Text or citation: <https://ovidsp.ovid.com/ovidweb.cgi?T=JS&CSC=Y&NEWS=N&PAGE=fulltext&D=pmnm&AN=37303911>

Postlbauer, A., Helm, C., Grose, C. S. (2022). Educational inequality and covid-19: who takes advantage of summer
schools and other remedial measures?: a latent mediation model analysis based on representative data from austrian
parents of school-aged children..  Zeitschrift Fr Bildungsforschung, , 12(2), 407-436.
<https://dx.doi.org/10.1007/s35834-022-00356-4>
 
Link to the Ovid Full Text or citation: <https://ovidsp.ovid.com/ovidweb.cgi?T=JS&CSC=Y&NEWS=N&PAGE=fulltext&D=pmnm&AN=37521430>
 
Quinn, E., Hsiao, K. H., Johnstone, T., Gomez, M., Parasuraman, A., Ingleton, A., Hirst, N., Najjar, Z., Gupta, L.
(2023). Protecting older adult residents in care facilities against influenza and covid-19 using the influenza
communication, advice and reporting (flucare) app: prospective cohort mixed methods study..  JMIR Formative Research, ,
7, e38080. <https://dx.doi.org/10.2196/38080>
 
Link to the Ovid Full Text or citation: <https://ovidsp.ovid.com/ovidweb.cgi?T=JS&CSC=Y&NEWS=N&PAGE=fulltext&D=pmnm&AN=36763638>
 
Rahman, M. M., Chisty, M. A., Sakib, M. S., Quader, M. A., Shobuj, I. A., Alam, M. A., Halim, M. A., Rahman, F. (2021).
Status and perception toward the covid-19 vaccine: a cross-sectional online survey among adult population of
bangladesh..  Health Science Reports, , 4(4), e451. <https://dx.doi.org/10.1002/hsr2.451>
 
Link to the Ovid Full Text or citation: <https://ovidsp.ovid.com/ovidweb.cgi?T=JS&CSC=Y&NEWS=N&PAGE=fulltext&D=pmnm&AN=34938896>
 
Ratnayake, R., Rawashdeh, F., AbuAlRub, R., Al-Ali, N., Fawad, M., Bani Hani, M., Zoubi, S., Goyal, R., Al-Amire, K.,
Mahmoud, R., AlMaaitah, R., Parmar, P. K. (2022). Rapidly adapted community health strategies to prevent treatment
interruption and improve covid-19 detection for syrian refugees and the host population with hypertension and diabetes
in jordan..  International Health, , . <https://dx.doi.org/10.1093/inthealth/ihac083>
 
Link to the Ovid Full Text or citation: <https://ovidsp.ovid.com/ovidweb.cgi?T=JS&CSC=Y&NEWS=N&PAGE=fulltext&D=medp&AN=36576492>
 
Rendell, N., Sheel, M. (2022). Expert perspectives on priorities for supporting health security in the pacific region
through health systems strengthening..  PLOS Global Public Health, , 2(9), e0000529.
<https://dx.doi.org/10.1371/journal.pgph.0000529>
 
Link to the Ovid Full Text or citation: <https://ovidsp.ovid.com/ovidweb.cgi?T=JS&CSC=Y&NEWS=N&PAGE=fulltext&D=pmnm&AN=36962587>


Romero-Ortuno, R., Jennings, G., Xue, F., Duggan, E., Gormley, J., Monaghan, A. (2022). Predictors of submaximal
exercise test attainment in adults reporting long covid symptoms..  Journal of Clinical Medicine, , 11(9), .
<https://dx.doi.org/10.3390/jcm11092376>
 
Link to the Ovid Full Text or citation: <https://ovidsp.ovid.com/ovidweb.cgi?T=JS&CSC=Y&NEWS=N&PAGE=fulltext&D=pmnm&AN=35566502>


Santus, P., Radovanovic, D., Gismondo, M. R., Rimoldi, S. G., Lombardi, A., Danzo, F., Gori, A., Antinori, S.,
Rizzardini, G. (2023). Respiratory syncytial virus burden and risk factors for severe disease in patients presenting to
the emergency department with flu-like symptoms or acute respiratory failure..  Respiratory Medicine, , 218, 107404.
<https://dx.doi.org/10.1016/j.rmed.2023.107404>
 
Link to the Ovid Full Text or citation: <https://ovidsp.ovid.com/ovidweb.cgi?T=JS&CSC=Y&NEWS=N&PAGE=fulltext&D=medp&AN=37683776>
 
Schneider, E., Hopf, D., Eckstein, M., Scheele, D., Aguilar-Raab, C., Herpertz, S. C., Grinevich, V., Ditzen, B. (2023).
Stress during the covid-19 pandemic moderates pain perception and momentary oxytocin levels..  Journal of Clinical
Medicine, , 12(6), . <https://dx.doi.org/10.3390/jcm12062333>
 
Link to the Ovid Full Text or citation: <https://ovidsp.ovid.com/ovidweb.cgi?T=JS&CSC=Y&NEWS=N&PAGE=fulltext&D=pmnm&AN=36983333>

Schroder, J., Bauerle, A., Jahre, L. M., Skoda, E. M., Stettner, M., Kleinschnitz, C., Teufel, M., Dinse, H. (2023).
Acceptance, drivers, and barriers to use ehealth interventions in patients with post-covid-19 syndrome for management of
post-covid-19 symptoms: a cross-sectional study..  Therapeutic Advances in Neurological Disorders, , 16,
17562864231175730. <https://dx.doi.org/10.1177/17562864231175730>
 
Link to the Ovid Full Text or citation: <https://ovidsp.ovid.com/ovidweb.cgi?T=JS&CSC=Y&NEWS=N&PAGE=fulltext&D=pmnm&AN=37255668>

Sekkarie, A., Woodruff, R., Whitaker, M., Kramer, M. R., Zapata, L. B., Ellington, S. R., Meaney-Delman, D. M., Pham,
H., Patel, K., Taylor, C. A., Chai, S. J., Kawasaki, B., Meek, J., Openo, K. P., Weigel, A., Leegwater, L.,
Como-Sabetti, K., Ropp, S. L., Muse, A., Bennett, N. M., Billing, L. M., Sutton, M., Talbot, H. K., Hill, M., Havers, F.
P., COVID-19-Associated Hospitalization Surveillance Network COVID-NET Surveillance Team (2022). Characteristics and
treatment of hospitalized pregnant women with covid-19..  American Journal of Obstetrics & Gynecology MFM, , 4(6),
100715. <https://dx.doi.org/10.1016/j.ajogmf.2022.100715>
 
Link to the Ovid Full Text or citation: <https://ovidsp.ovid.com/ovidweb.cgi?T=JS&CSC=Y&NEWS=N&PAGE=fulltext&D=medp&AN=35970493>
 
Setianingrum, E. L. S., Lidia, K., Ratu, K., Teron, S. E. (2023). Assessment of post-vaccination immunologic responses
in inactivated virus covid-19 respondents..  Indian Journal of Community Medicine, , 48(2), 357-360.
<https://dx.doi.org/10.4103/ijcm.ijcm_477_22>
 
Link to the Ovid Full Text or citation: <https://ovidsp.ovid.com/ovidweb.cgi?T=JS&CSC=Y&NEWS=N&PAGE=fulltext&D=pmnm&AN=37323736>

Shen, K., Kejriwal, M. (2023). Quantifying covid-19 policy impacts on subjective well-being during the early phase of
the pandemic: a cross-sectional analysis of united states survey data from march to august 2020..  PLoS ONE [Electronic
Resource], , 18(9), e0291494. <https://dx.doi.org/10.1371/journal.pone.0291494>
 
Link to the Ovid Full Text or citation: <https://ovidsp.ovid.com/ovidweb.cgi?T=JS&CSC=Y&NEWS=N&PAGE=fulltext&D=prem&AN=37733714>
 
Shieh, D., Sevilla, M., Palmeri, A., Ly, A. H., Shi, J. M., Berringer, C., Resurreccion, J. (2023). The shieh score as a
risk assessment instrument for reducing hospital-acquired pressure injuries: a prospective cohort study..  Journal of
Wound, Ostomy, & Continence Nursing, , 50(5), 375-380. <https://dx.doi.org/10.1097/WON.0000000000000997>
 
Link to the Ovid Full Text or citation: <https://ovidsp.ovid.com/ovidweb.cgi?T=JS&CSC=Y&NEWS=N&PAGE=fulltext&D=pmnm&AN=37467392>
 
Singh, J., Arora, R., Rawat, V., Singh, V., Goyal, S., Joshi, L. (2023). Simplified screening and referral protocol for
sinonasal mucormycosis in post covid-19 patients..  Bulletin of the National Research Centre (Cairo), , 47(1), 58.
<https://dx.doi.org/10.1186/s42269-023-01032-x>
 
Link to the Ovid Full Text or citation: <https://ovidsp.ovid.com/ovidweb.cgi?T=JS&CSC=Y&NEWS=N&PAGE=fulltext&D=pmnm&AN=37128189>
 
Sisay, M. M., Montesinos-Guevara, C., Osman, A. K., Saraswati, P. W., Tilahun, B., Ayele, T. A., Ahmadizar, F., Duran,
C. E., Sturkenboom, M. C. J. M., van de Ven, P., Weibel, D. (2023). Covid-19 vaccine safety monitoring studies in low-
and middle-income countries (lmics)-a systematic review of study designs and methods..  101629355, , 11(6), .
<https://dx.doi.org/10.3390/vaccines11061035>
 
Link to the Ovid Full Text or citation: <https://ovidsp.ovid.com/ovidweb.cgi?T=JS&CSC=Y&NEWS=N&PAGE=fulltext&D=pmnm&AN=37376424>


Song, J. Y., Choi, W. S., Heo, J. Y., Kim, E. J., Lee, J. S., Jung, D. S., Kim, S. W., Park, K. H., Eom, J. S., Jeong,
S. J., Lee, J., Kwon, K. T., Choi, H. J., Sohn, J. W., Kim, Y. K., Yoo, B. W., Jang, I. J., Capeding, M. Z., Roman, F.,
Breuer, T., Wysocki, P., Carter, L., Sahastrabuddhe, S., Song, M., D'Cor, N., Kim, H., Ryu, J. H., Lee, S. J., Park, Y.
W., Cheong, H. J., GBP510/AS03 study group (2023). Immunogenicity and safety of sars-cov-2 recombinant protein
nanoparticle vaccine gbp510 adjuvanted with as03: interim results of a randomised, active-controlled, observer-blinded,
phase 3 trial..  EClinicalMedicine, , 64, 102140. <https://dx.doi.org/10.1016/j.eclinm.2023.102140>
 
Link to the Ovid Full Text or citation: <https://ovidsp.ovid.com/ovidweb.cgi?T=JS&CSC=Y&NEWS=N&PAGE=fulltext&D=pmnm&AN=37711219>

Stehlin, F., Mahdi-Aljedani, R., Canton, L., Monzambani-Banderet, V., Miauton, A., Girard, C., Kammermann, K., Meylan,
S., Ribi, C., Harr, T., Yerly, D., Muller, Y. D. (2022). Intradermal testing with covid-19 mrna vaccines predicts
tolerance..  Frontiers in Allergy, , 3, 818049. <https://dx.doi.org/10.3389/falgy.2022.818049>
 
Link to the Ovid Full Text or citation: <https://ovidsp.ovid.com/ovidweb.cgi?T=JS&CSC=Y&NEWS=N&PAGE=fulltext&D=pmnm&AN=36238929>
 
Stemerman, R., Bunning, T., Grover, J., Kitzmiller, R., Patel, M. D. (2021). Identifying patient phenotype cohorts using
prehospital electronic health record data..  Prehospital Emergency Care, , 1-14.
<https://dx.doi.org/10.1080/10903127.2020.1859658>
 
Link to the Ovid Full Text or citation: <https://ovidsp.ovid.com/ovidweb.cgi?T=JS&CSC=Y&NEWS=N&PAGE=fulltext&D=medp&AN=33315497>
 
Stickler, K., Morrone, B., Brown, A., Franks, A. M., Johnson, P., Lacmichanne, R., Rupp, D. (2023). Analysis of a
division i sports medicine program's covid-19 protocol: identifying factors that predict testing demands and positive
rates during a pandemic..  Clinical Journal of Sport Medicine, , . <https://dx.doi.org/10.1097/JSM.0000000000001176>
 
Link to the Ovid Full Text or citation: <https://ovidsp.ovid.com/ovidweb.cgi?T=JS&CSC=Y&NEWS=N&PAGE=fulltext&D=medp&AN=37432329>
 
Tahir, M. A., Khan, M. A., Ikram, A., Chaudhry, T. H., Jabeen, A., Quddous, A., Haq, I. U. (2023). Assessment of
infection prevention and control (ipc) implementation and strategies used for ipc preparedness at facility level in
underdeveloped areas of pakistan..  Infection & Drug Resistance, , 16, 1997-2006. <https://dx.doi.org/10.2147/IDR.S399830>
 
Link to the Ovid Full Text or citation: <https://ovidsp.ovid.com/ovidweb.cgi?T=JS&CSC=Y&NEWS=N&PAGE=fulltext&D=pmnm&AN=37038477>

Teklemariam, Z., Feleke, D., Abdurahman, A., Alemayehu, A., Demissie, A., Tufa, A., Sherefa, N., Mohammed, A., Brhane,
M., Bogale, K. (2023). Evaluation of the performance of abbott panbio tm covid-19 antigen rapid diagnostic test for the
detection of severe acute respiratory syndrome coronavirus 2 at harar, eastern ethiopia..  Frontiers in Medicine, , 10,
1135027. <https://dx.doi.org/10.3389/fmed.2023.1135027>
 
Link to the Ovid Full Text or citation: <https://ovidsp.ovid.com/ovidweb.cgi?T=JS&CSC=Y&NEWS=N&PAGE=fulltext&D=pmnm&AN=37324158>


Thompson, J. A., Hersch, D., Kasozi, R. N., Miner, M. H., Adam, P. (2023). Disparities in offering enrollment in remote
patient monitoring for covid-19..  Telemedicine Journal & E-Health, , . <https://dx.doi.org/10.1089/tmj.2023.0150>
 
Link to the Ovid Full Text or citation: <https://ovidsp.ovid.com/ovidweb.cgi?T=JS&CSC=Y&NEWS=N&PAGE=fulltext&D=medp&AN=37707989>
 
Vigotti, F. N., Di Benedetto, C., Fop, F., Bianco, S., Bilucaglia, D., Cesano, G. (2023). Lung ultrasonography performed
by nephrologist: covid-19 as an opportunity to reveal ultrasound's full potential and usefulness in the dialysis room..
Clinical Kidney Journal, , 16(3), 541-548. <https://dx.doi.org/10.1093/ckj/sfac250>
 
Link to the Ovid Full Text or citation: <https://ovidsp.ovid.com/ovidweb.cgi?T=JS&CSC=Y&NEWS=N&PAGE=fulltext&D=pmnm&AN=36865007>

Ward, H., Atchison, C., Whitaker, M., Davies, B., Ashby, D., Darzi, A., Chadeau-Hyam, M., Riley, S., Donnelly, C. A.,
Barclay, W., Cooke, G. S., Elliott, P. (2023). Design and implementation of a national program to monitor the prevalence
of sars-cov-2 igg antibodies in england using self-testing: the react-2 study..  American Journal of Public Health, ,
e1-e9. <https://dx.doi.org/10.2105/AJPH.2023.307381>
 
Link to the Ovid Full Text or citation: <https://ovidsp.ovid.com/ovidweb.cgi?T=JS&CSC=Y&NEWS=N&PAGE=fulltext&D=medp&AN=37733993>
 
OpenSAFELY Collaborative, Williamson, E. J., Tazare, J., Bhaskaran, K., McDonald, H. I., Walker, A. J., Tomlinson, L.,
Wing, K., Bacon, S., Bates, C., Curtis, H. J., Forbes, H. J., Minassian, C., Morton, C. E., Nightingale, E., Mehrkar,
A., Evans, D., Nicholson, B. D., Leon, D. A., Inglesby, P., MacKenna, B., Davies, N. G., DeVito, N. J., Drysdale, H.,
Cockburn, J., Hulme, W. J., Morley, J., Douglas, I., Rentsch, C. T., Mathur, R., Wong, A., Schultze, A., Croker, R.,
Parry, J., Hester, F., Harper, S., Grieve, R., Harrison, D. A., Steyerberg, E. W., Eggo, R. M., Diaz-Ordaz, K., Keogh,
R., Evans, S. J. W., Smeeth, L., Goldacre, B. (2022). Comparison of methods for predicting covid-19-related death in the
general population using the opensafely platform..  Diagnostic and Prognostic Research, , 6(1), 6.
<https://dx.doi.org/10.1186/s41512-022-00120-2>
 
Link to the Ovid Full Text or citation: <https://ovidsp.ovid.com/ovidweb.cgi?T=JS&CSC=Y&NEWS=N&PAGE=fulltext&D=pmnm&AN=35197114>


Wolf, L., Buschini, L., Logsdon, R. (2023). Family nurse practitioner students' readiness for board certification
utilizing a full board review course and simulation..  SAGE Open Nursing, , 9, 23779608231186031.
<https://dx.doi.org/10.1177/23779608231186031>
 
Link to the Ovid Full Text or citation: <https://ovidsp.ovid.com/ovidweb.cgi?T=JS&CSC=Y&NEWS=N&PAGE=fulltext&D=pmnm&AN=37425285>


Wu, H. H., Su, C. H., Chien, L. J., Tseng, S. H., Chang, S. C. (2023). Coronavirus disease 2019 (covid-19) universal
admission screening in patients and companions in taiwan from may 2021 to june 2022: a nationwide multicenter study..
Infection Control & Hospital Epidemiology, , 1-7. <https://dx.doi.org/10.1017/ice.2023.144>
 
Link to the Ovid Full Text or citation: <https://ovidsp.ovid.com/ovidweb.cgi?T=JS&CSC=Y&NEWS=N&PAGE=fulltext&D=medp&AN=37462097>
 
Yohannes, S., Seam, N., Sun, J., McAlduff, J., Thorne, J. L., Lara, S. B., Keller, M. (2023). Impact of an early warning
system protocol, for patients admitted to the medical floors with sars-cov2 pneumonia, on icu admission..  Clinical
medicine insights. Circulatory, respiratory and pulmonary medicine [electronic resource], , 17, 11795484231156755.
<https://dx.doi.org/10.1177/11795484231156755>
 
Link to the Ovid Full Text or citation: <https://ovidsp.ovid.com/ovidweb.cgi?T=JS&CSC=Y&NEWS=N&PAGE=fulltext&D=pmnm&AN=36968975>
 
Zahra, A., Luijken, K., Abbink, E. J., van den Berg, J. M., Blom, M. T., Elders, P., Festen, J., Gussekloo, J., Joling,
K. J., Melis, R., Mooijaart, S., Peters, J. B., Polinder-Bos, H. A., van Raaij, B. F. M., Smorenberg, A., la Roi-Teeuw,
H. M., Moons, K. G. M., van Smeden, M., COOP Consortium (2023). A study protocol of external validation of eight
covid-19 prognostic models for predicting mortality risk in older populations in a hospital, primary care, and nursing
home setting..  Diagnostic and Prognostic Research, , 7(1), 8. <https://dx.doi.org/10.1186/s41512-023-00144-2>
 
Link to the Ovid Full Text or citation: <https://ovidsp.ovid.com/ovidweb.cgi?T=JS&CSC=Y&NEWS=N&PAGE=fulltext&D=pmnm&AN=37013651>
